# DFT‐Guided Design of a Multi‐Enzyme Mimetic High‐Entropy Nanozyme for Cascaded Glutathione Detection and Point‐of‐Care Testing

**DOI:** 10.1002/advs.202515765

**Published:** 2025-11-05

**Authors:** Hua Lin, Li Ke, Shuran Wang, Ruoke Li, Shengmin Zhou, Yueling Liu, Huan Pang

**Affiliations:** ^1^ State Key Laboratory of Bioreactor Engineering School of Biotechnology East China University of Science and Technology Shanghai 200237 P. R. China; ^2^ School of Chemistry and Chemical Engineering Yangzhou University Jiangsu 225009 P. R. China

**Keywords:** colorimetric, glutathione, high‐entropy nanozyme, microfluidic paper, Prussian blue analogues

## Abstract

Despite of the various advantages of Prussian blue analogs (PBA), most of the previous studies rely on conventional empirical and trial‐and‐error methods for identifying the effective catalytic activity. Theory‐guided design using predictive models is leading a new revolution in the field of nanozymes, which has yet to focus on PBA. Herein, a series of PBAs, including binary, ternary, quaternary, quinary, and high‐entropy, are first investigated by density functional theory (DFT) calculations. Both DFT and experimental results prove the superior catalytic activity of high‐entropy than medium‐ and low‐entropy PBAs, mainly owing to the enhanced *d*‐band centers near the Fermi energy level (*E*_F_). The proposed high‐entropy PBA oxide (HEO, MnCoNiCuZnFe) demonstrates multi‐enzymatic activities. To achieve the colorimetric detection of glutathione (GSH), a colorimetric system with 3,3′,5,5′‐tetramethylbenzidine (TMB) and H_2_O_2_ is employed and exhibits a low detection limit, excellent selectivity, remarkable reusability, and long‐term stability. Moreover, DFT calculations elucidate the electronic structure by revealing the Gibbs free energy of H_2_O_2_ dissociation on individual transition‐metal sites. Additionally, a microfluidic paper‐based analytical device for the point‐of‐care testing (POCT) of GSH is successfully developed. This study not only provides a rational design strategy for multi‐metallic nanomaterials, but also expands the application of high‐entropy PBA nanozymes.

## Graphical abstract

Guided by density functional theory (DFT) calculations, a MnCoNiCuZnFe‐based Prussian blue analog (HEO) is screened out. It exhibits superior peroxidase (POD)‐like activity compared to noble metal‐incorporated high‐entropy nanozymes together with excellent oxidase (OXD) and glutathione oxidase (GSHOx)‐like activities. To achieve the point‐of‐care testing (POCT), a ready‐to‐use dumbbell‐shaped microfluidic paper‐based analytical device is successfully developed for colorimetric glutathione (GSH) detection.

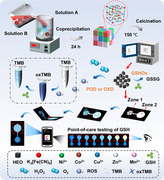

## 1 Introduction

Nanozymes, a category of nanomaterials exhibiting enzyme‐like catalytic properties, offer advantages such as ease of storage, high catalytic efficiency, robust stability, and excellent reusability.^[^ ^1^ ^]^ Since Yan and coworkers first discovered Fe_3_O_4_ nanoparticles with intrinsic peroxidase (POD)‐like activity,^[^ ^2^ ^]^ metals, non‐metals, and metal–organic framework (MOF) based materials have been widely applied in biosensing, electrocatalysis, therapeutics, and environmental protection.^[^ ^3^, ^4^, ^5^, ^6^, ^7^, ^8^ ^]^ Despite of the highly catalytic efficiency of nanozyme with noble metals doping or heterojunctions, or a combination of both, the complex synthesis and scarce reserves impede their widespread application. Transition metal‐based nanomaterials are renowned for their diverse oxidation states, abundant active sites, and low cost, which is undoubtedly a promising candidate for nanozyme research.

Prussian blue analogs (PBA), a family of perovskite‐like nanomaterials bridged by cyanide groups and composed of transition metals, hold great promise owing to their unique structural and functional properties.^[^ ^9^, ^10^ ^]^ Specifically, their high specific surface area and rich active sites enhance catalytic performance, while their porous framework structure facilitates efficient mass transport and reaction kinetics optimization.^[^ ^11^, ^12^ ^]^ Recent advancements in PBA‐based nanozymes have demonstrated their potential in mimicking peroxidase, catalase, and superoxide dismutase activities.^[^ ^13^, ^14^, ^15^, ^16^ ^]^ The introduction of high‐entropy into PBA has introduced new research possibilities in electrocatalysis, energy storage, and ion batteries.^[^ ^17^, ^18^, ^19^, ^20^ ^]^ High‐entropy materials are formally defined as entropy‐stabilized compounds comprising five or more cationic species in one system.^[21,22^^]^ The high‐entropy effect, elemental diversity, and cocktail effect collectively contribute to their exceptional structural stability and tunable functional properties, positioning it as a promising class of materials for advanced applications.^[^ ^23^, ^24^, ^25^ ^]^ Conventional high‐entropy materials were mostly designed based on trial‐and‐error synthesis and batch‐by‐batch validation strategies to screen out nanozymes with high catalytic activity, which was time‐consuming and ineffective. Given the variety of PBA with different compositions, structures, and morphologies, it is essential to establish multi‐metallic nanocatalytic platforms based on a clear theoretical guidance to expedite the discovery of nanozymes.

Recently, as research on nanozymes has deepened, scientists have integrated experimental investigations with computer simulations that explore the structure and behavior at atomic and molecular scales, as well as the physical and chemical properties of materials.^[^ ^26^ ^]^ The most widely used first‐principles approach is based on density functional theory (DFT). It enables the construction of framework models that closely resemble real structures, aids in identifying catalytic active sites and mechanisms on the surface, explains the factors influencing catalysis, and provides theoretical guidance for the rational design of nanozymes.^[^ ^27^, ^28^ ^]^ For instance, DFT calculations has been effectively used to identify two optimal dual‐atom sites (Fe/Co) from fourth‐period transition metals for high‐efficiency oxidase (OXD)‐like enzymes, demonstrating its potential in accelerating nanzozyme discovery.^[^ ^29^ ^]^ However, to the best of our knowledge, it has not been utilized in PBA nanozymes, whose cyanide‐bridged metal centers and mixed valence states pose significant computational challenges.

In order to rationally design PBA nanozymes, DFT was employed to forecast the catalytic activity of various PBA nanozymes, including binary, ternary, quaternary, quinary, and high‐entropy. Both DFT calculations and experimental results revealed that an increase in component diversity caused an upward shift in the *d*‐band center of PBA nanomaterials, strengthening the adsorption of oxygen intermediates and consequently improving the catalytic activity. The resulting high‐entropy oxide (HEO, MnCoNiCuZnFe) exhibited superior POD‐like activity than medium‐ and low‐ entropy PBA oxides. Utilizing advanced characterization techniques in conjunction with DFT calculations, the synergistic effects of HEO on catalytic performance were rigorously elucidated. Besides, the OXD‐ and glutathione oxidase (GSHOx)‐like catalytic activities, along with their underlying mechanisms, were systematically investigated to provide a comprehensive understanding of the functionality. As a proof‐of‐concept, this study integrated HEO with the cascade catalysis system of 3,3′,5,5′‐tetramethylbenzidine (TMB) and hydrogen peroxide (H_2_O_2_) to devise a sensitive and colorimetric analytical platform for detecting glutathione (GSH). The proposed HEO nanozyme catalyzed H_2_O_2_ to generate reactive oxygen species (ROS), which oxidized the colorless TMB to blue oxTMB. GSH scavenged the ROS and led to the termination of TMB oxidation. Furthermore, to realize the point‐of‐care testing (POCT) of GSH, a microfluidic paper‐based analytical device (µPAD) was employed, offering a highly practical, cost‐effectiveness, and high‐throughput solution for on‐site analysis. The µPAD used wax printing to create hydrophobic barriers and designed optimized reaction and detection zones. Through a straightforward folding process, the flow path of reaction liquids was precisely controlled after the introduction of reagents and samples, ensuring both reliability and reproducibility. This study not only provides a new approach for the rational design and regulation of multi‐metallic nanomaterials with high catalytic activity, but also broadens the potential applications of transition‐metal high‐entropy nanozymes.

## 2 Results and Discussion

### 2.1 Density Functional Theory Calculations

DFT is a technique used to examine the electronic structure of multi‐electron systems by performing precise calculations of electron density distributions. Owing to its favorable balance of predictive capability, computational efficiency, and broad applicability, DFT has become a widely employed method in materials science, catalytic chemistry, quantum chemical simulations, and related fields.^[^ ^30^ ^]^ DFT calculations were utilized first to screen a series of PBAs with different transition metal elements as potential catalytic active sites, including of binary (ZnFe), ternary (CuZnFe), quaternary (NiCuZnFe), quinary (CoNiCuZnFe), and high‐entropy (MnCoNiCuZnFe), with the goal of promoting the theory‐driven synthesis of nanozymes. All selected metals share the same charge and have similar ionic radii, which facilitates randomly occupying the same metal sites within the PBA lattice.^[^ ^19^ ^]^ This homogeneity ensures the formation of a solid solution without phase separation, contributing to the enhanced structural stability. Besides, they are earth‐abundant, cost‐effective, and low‐toxic transition metals, making them suitable for the development of low‐cost, large‐scale, and environmentally friendly nanozymes.

**Figure 1:**
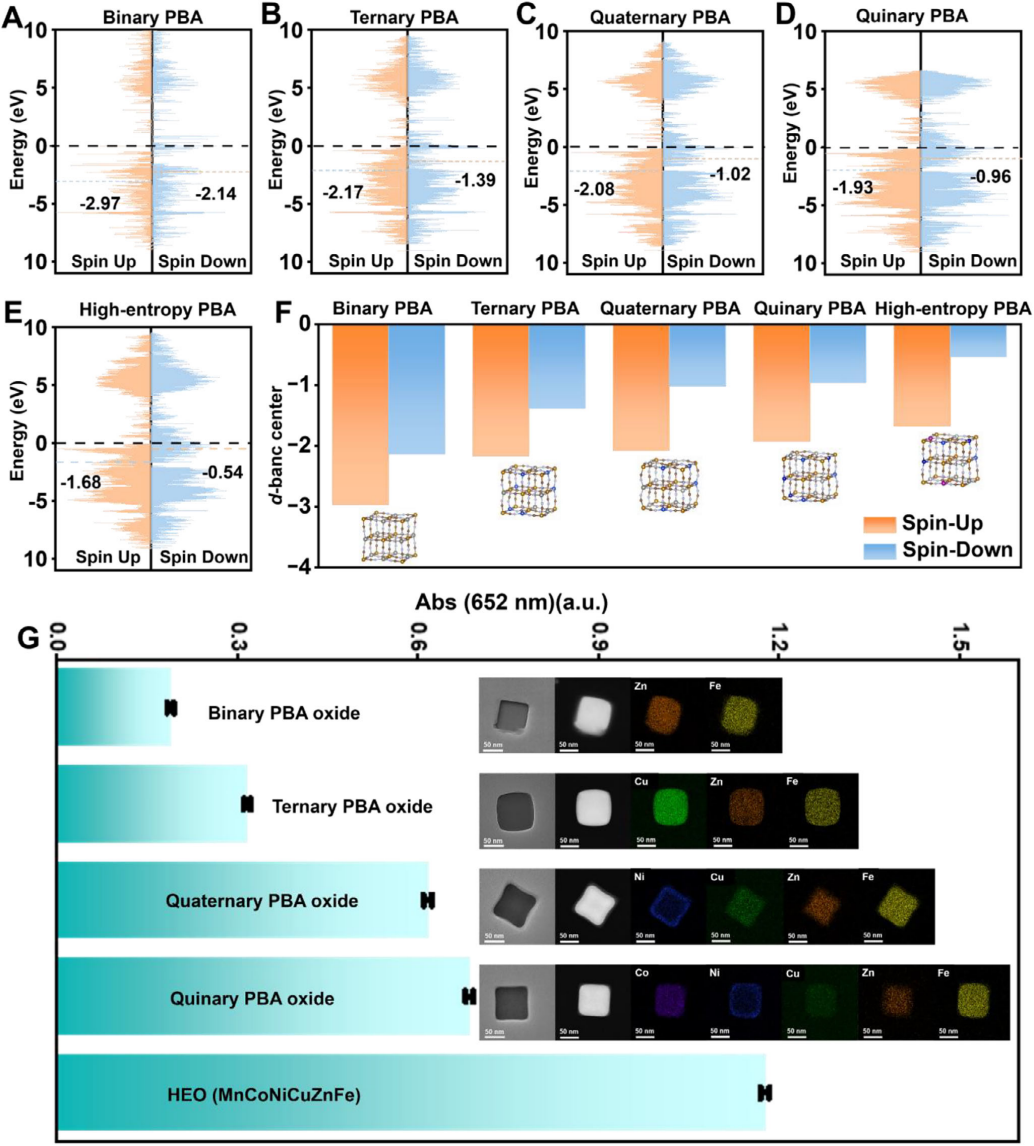
Theoretical and experimental comparison of a series of PBA nanozymes with different compositions. Total density of states (TDOS) for A) binary, B) ternary, C) quaternary, D) quinary, and E) high‐entropy PBAs. F) Comparison of *d*‐band centers (spin‐up and spin‐down) for binary, ternary, quaternary, quinary, and high‐entropy PBAs, together with their atomic model schematics obtained from DFT calculations as insets. G) Maximum absorbance at 652 nm (A_652_) of the POD‐like activity for various PBA oxides, including binary, ternary, quaternary, quinary, and high‐entropy (HEO) (*n* = 3), together with corresponding TEM and elemental mapping images as insets. Scale bar: 50 nm.

Prior to the initiation of all experimental procedures, the total density of states (TDOS) for binary, ternary, quaternary, quinary, and high‐entropy PBAs was meticulously computed utilizing first‐principles theory. This computational approach was necessitated by the well‐established correlation between the surface atomic composition of nanozymes and the adsorption of substrates, as well as electron transfer during catalytic processes, which collectively modulate the catalytic activity.^[^ ^31^ ^]^ **Figure** **1**A–E exhibits a comprehensive illustration of TDOS for ZnFe), ternary (CuZnFe), quaternary (NiCuZnFe), quinary (CoNiCuZnFe), and high‐entropy (MnCoNiCuZnFe) PBAs. For comparison, a detailed representation of the spin‐up and spin‐down *d*‐band centers is shown in Figure 1F alongside their corresponding atomic models as insets. Among all the PBAs investigated, the *d*‐band centers gradually shift to the Fermi level (*E*_F_) with increasing amount of metal element species. The high‐entropy PBA exhibits a *d*‐band center closest to the *E*_F_, with spin‐up and spin‐down *d*‐band centers positioned at −1.68 and −0.54, respectively. It illustrates the strongest interaction between the nanozyme and reaction intermediates, facilitating the catalytic reaction.^[^ ^32^ ^]^ Consequently, the DFT calculations predict that the high‐entropy PBA (MnCoNiCuZnFe) have the highest catalytic activity.

In comparison, we also analyzed the electronic structure of these PBAs without cyanide bridging groups (simplified PBA models). Their TDOS for ZnFe, CuZnFe, NiCuZnFe, CoNiCuZnFe, and MnCoNiCuZnFe nanozymes is exhibited in Figure S2 (Supporting Information). In general, a substantial downward shift is observed in all the *d*‐band centers in these simplified single‐atom models compared to Figure 1F. The probable reason is the cyanide groups mediating efficient electron delocalization across the metal‐cyanide‐metal framework, achieving the synergistic effect. The results confirm the pivotal role of the cyanide bridge in modulating the electronic properties of the metal centers in PBAs, thereby influencing their catalytic activity.^[^ ^33^ ^]^ The inclusion of the full bridge in our PBA models, contrasting with simplified PBA models, allows us to more accurately capture this synergistic effect, which is a marked improvement in theory‐guided design strategy.

**Figure 2:**
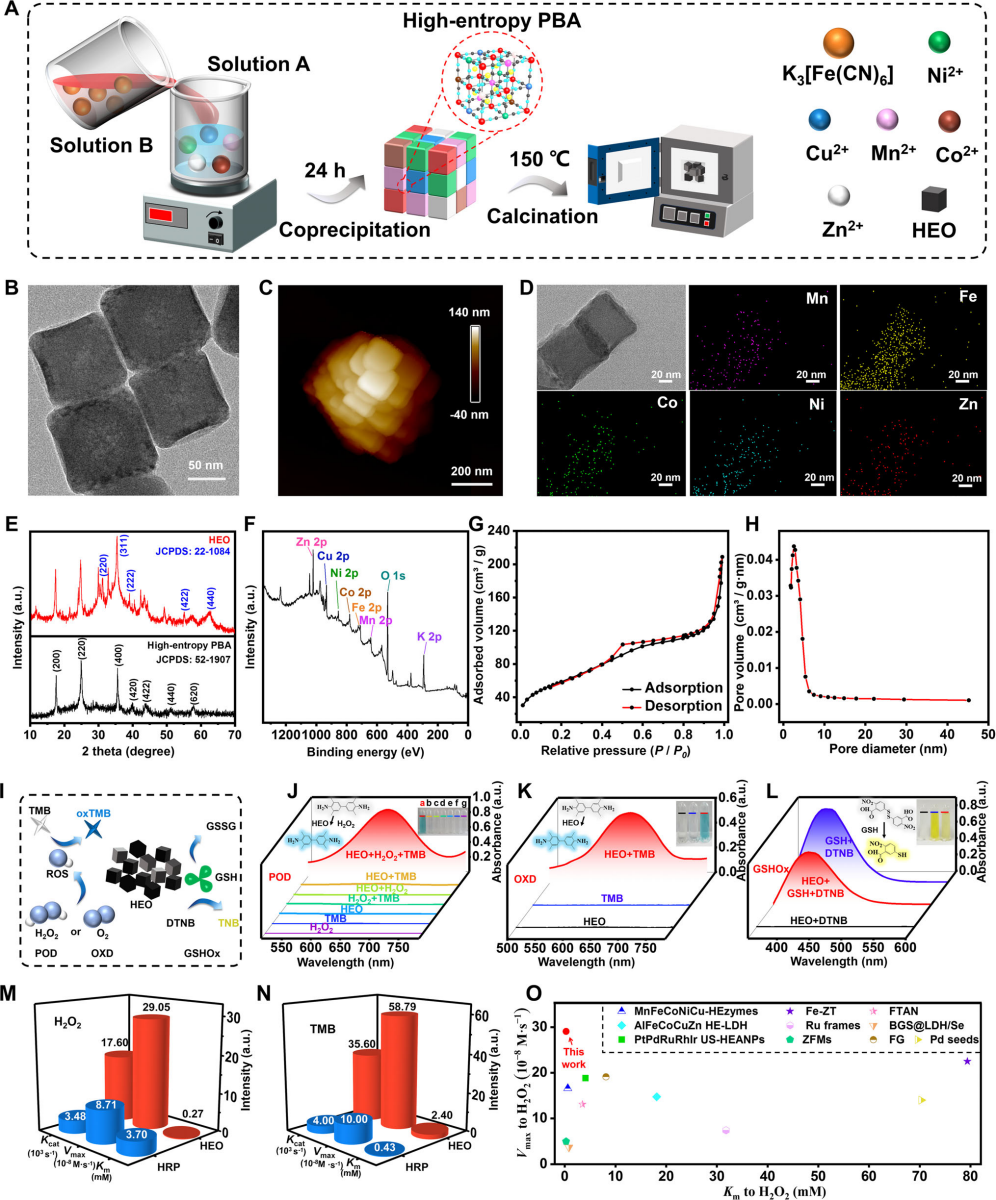
Synthesis procedure, structural characterization, and multi‐enzyme mimicking activities of HEO. A) Schematic illustration of the synthetic process. B) STEM (Scale bar: 50 nm) and C) AFM (Scale bar: 200 nm) images, as well as D) elemental mapping analysis (Scale bar: 20 nm), including Mn, Fe, Co, Ni, and Zn. E) XRD patterns of HEO (red line) and high‐entropy PBA (black line). F) XPS spectrum of HEO including of Zn 2p, Cu 2p, Ni 2p, Co 2p, Fe 2p, Mn 2p, O 1s, and K 2p. G) Nitrogen adsorption‐desorption isotherm and H) corresponding pore size distribution curve. I) Schematic illustration of the catalytic mechanism of HEO. J) UV–vis absorption spectra of (a) HEO/TMB/H_2_O_2_ (red), (b) HEO/TMB (yellow), (c) HEO/H_2_O_2_ (light green), (d) H_2_O_2_/TMB (deep green), (e) HEO (light blue), (f) TMB (dark blue), and (g) H_2_O_2_ (purple) in NaAc‐HAc buffer (pH 4.00) under N_2_ atmosphere to investigate the POD‐like activity. K) UV–vis absorption spectra of HEO/TMB (red), TMB (blue), and HEO (black) in NaAc‐HAc buffer (pH 4.00) to study the OXD‐like activity. L) UV–vis absorbance spectra of GSH/DTNB (blue), HEO/GSH/DTNB (red), and HEO/DTNB (black) in PBS buffer (pH 6.50) to assess the GSHOx‐like activity. Comparative study of kinetic parameters between HEO and HRP, including *K*_m_, *V*_max_, and *K*_cat_ to M) H_2_O_2_ and N) TMB. O) Comparison of kinetic parameters *K*_m_ and *V*_max_ for HEO to H_2_O_2_ with previously reported simulated POD‐like nanozymes.^[^ ^44^, ^48^, ^49^, ^50^, ^51^, ^52^, ^53^, ^54^, ^55^, ^56^ ^]^

To validate the accuracy of the DFT calculations, the POD‐like activity of these PBA oxides was investigated. Prior to the POD‐like activity assay, the binary, ternary, quaternary, quinary, and high‐entropy oxides were characterized by transmission electron microscopy (TEM) and elemental mapping analysis (Figure 1G inset and **Figure** **2**D). All synthesized PBA oxides exhibit well‐defined cubic morphology with highly uniform element distributions, confirming the successful formation of these multi‐component systems. Afterward, these PBA oxides was introduced into a mixed solution containing TMB and H_2_O_2_ in sodium acetate‐acetic acid (NaAc‐HAc) buffer to facilitate the catalytic reactions. The colorless TMB can be oxidized to blue oxTMB with a characteristic absorbance at 652 nm (A_652_). As depicted in Figure 1G, the A_652_ enhanced from binary to high‐entropy oxides with increasing transition metal species, suggesting the improved POD‐like activity. Among various nanozymes, the HEO (MnCoNiCuZnFe) enjoys the highest POD‐like activity, proving the accuracy of the above theoretical calculations. Therefore, building upon the DFT calculations and experimental results, a highly efficient high‐entropy PBA nanozyme with a composition of MnCoNiCuZnFe is obtained.

### 2.2 Characterization of HEO

Five metal nitrates, including of Mn^2+^, Co^2+^, Ni^2+^, Cu^2+^, and Zn^2+^, together with potassium ferricyanide, are utilized to fabricate the proposed HEO via a simple co‐precipitation method with water as solvent and followed by calcination at 150 °C (Figure 2A). The morphological and structural properties of the HEO were examined using TEM and atomic force microscopy (AFM). The resulting TEM and AFM micrographs, present in Figure 2B,C, both demonstrated a cubic morphology. The dynamic light scattering (DLS) in Figure S3A (Supporting Information) displays an average size of ≈300 nm owing to the aggregation of HEO without ultrasonication, which is consistent to the AFM images in Figure 2C. Besides, the elemental mapping analysis in Figure 2D and Figure S3B (Supporting Information) reveals the uniform distribution of Mn, Fe, Co, Ni, and Zn, indicating the successful synthesis of HEO. The X‐ray diffraction (XRD) spectrum of HEO is exhibites in Figure 2E (red line), which was compared to that of high‐entropy PBA (black line). It displays peaks at 17.62°, 24.88°, 39.90°, 43.92°, and 57.80°, referring to crystal faces (200), (220), (420), (422), and (620) (Prussian blue, JCPDS 52–1907). These peaks appear in both high‐entropy PBA and HEO. The difference lies in the peaks at 30.38°, 35.48°, 37.69°, 55.31°, and 63.11° (red line in Figure 2E), which correspond to the (220), (311), (222), (422), and (440) crystal faces (JCPDS 22–1084), exhibiting characteristic magnetite peaks (Fe_3_O_4_).^[^ ^34^ ^]^ The peaks at 35.56° and 51.22° (black line in Figure 2E) correspond to crystal faces (400) and (440) (JCPDS 52–1907), which is ascribed to the characteristic peaks of cyanide‐bridged metal centers in high‐entropy PBA.^[^ ^35^ ^]^

Further characterization of the elemental valence of HEO was carried out through X‐ray photoelectron spectroscopy (XPS). As depicted in Figure 2F and Figure S4 (Supporting Information), the Mn 2p, Fe 2p, Co 2p, and Ni 2p spectra indicated the presence of the corresponding divalent and trivalent ions, whereas the Zn 2p and Cu 2p spectra suggested the presence of divalent species.^[^ ^36^, ^37^ ^]^ Besides, the gas pycnometer showed that the density of the HEO was determined to be 2.52 g g· cm^−3^. To evaluate the specific surface area and surface adsorption properties, Brunauer–Emmet–Teller (BET) analysis is conducted in Figure 2G. The nitrogen adsorption‐desorption isotherm exhibited a characteristic type IV curve.^[^ ^38^, ^39^ ^]^ Because of the presence of mesopores, capillary condensation of nitrogen occurred, resulting in a hysteresis loop in the relative pressure (*P/P_0_*) range of 0.40–0.90. The existence of some macropores led to a large amount of absorption at *P/P_0_* > 0.90.^[^ ^40^ ^]^ Based on the BET and Barrett–Joyner–Halenda (BJH) models, the specific surface area, pore volume, and average pore diameter of the HEO are 216.01 m^2^·g^−1^, 0.29 cm^3^·g^−1^, and 6.67 nm, respectively (Figure 2H).^[^ ^41^ ^]^ The abundant voids are beneficial to the rapid transfer of electrons, which is conducive to the redox ability of the HEO.^[^ ^42^, ^43^ ^]^

In order to investigate the multi‐enzyme activities of HEO, we use TMB as a chromogenic substrate for POD‐ and OXD‐like activities together with 5,5′‐dithiobis‐(2‐nitrobenzoic acid) (DTNB) for GSHOx‐like activity (Figure 2I). To evaluate the TMB oxidation caused by the HEO specifically, the absorbance spectra of different solutions consisting of H_2_O_2_ (purple line, g), TMB (dark blue line, f), HEO (light blue, e), TMB/H_2_O_2_ (dark green, d), HEO/H_2_O_2_ (light green, c), HEO/TMB (orange, b), and HEO/TMB/H_2_O_2_ (red, a) are compared in Figure 2J. It illustrates that only when H_2_O_2_, TMB, and HEO co‐existed, the maximum peak appeared at about A_652_, demonstrating the oxidation of TMB specifically by HEO. Besides, the corresponding reaction solution in tube a (Figure 2J inset) was blue, which is different from the colorless tube b–g. The result proves that the HEO could act a simulator mimicking horseradish peroxidase (HRP). We also investigated the effects of varying calcination temperatures, including 100, 150, 200, and 400 °C on the catalytic activity of HEO. Figure S5A (Supporting Information) shows that the HEO calcined at 150 °C had the maximum A_652_, showing the highest catalytic activity. This may be due to the incomplete decomposition of the PBA framework after calcination at 150 °C and the exposure of more active metal sites, thus fascinating the electron transfer rate.^[^ ^43^ ^]^ Further increasing the calcination temperature to 500 °C leads to the complete collapse of its morphology, as demonstrated by the AFM image in Figure S5B (Supporting Information). Accordingly, the significant decrease in activity after calcination at 400 °C is likely due to the partial collapse of the porous PBA oxide framework and the sintering of nanoparticles. To investigate the OXD‐like activity, the absorption spectrum of the HEO/TMB reaction system is illustrated in Figure 2K (red) together with the blue solution as inset. This is because the OXD‐like activity of HEO could catalyze the conversion of O_2_ to superoxide radical anions (·O_2_^−^), making the colorless TMB to blue oxTMB with a characteristic A_652_. In contrast, neither the HEO (black) nor TMB (blue) alone show absorption and the corresponding solutions remained colorless (insets in Figure 2K). Moreover, to evaluate the GSHOx‐like activity, the absorption spectra of GSH/DTNB (blue), HEO/GSH/DTNB (red), and HEO/DTNB (black) are exhibited in Figure 2L. Since the DTNB can react with GSH to form a yellow 2‐nitro‐5‐thiobenzoic acid (TNB) with an absorption peak at ≈412 nm (A_412_).^[^ ^44^ ^]^ After the introduction of HEO, the maximum A_412_ decreased (red) and the color of the solution faded from bright yellow to light yellow (insets in Figure 2L). The results confirm the excellent multi‐enzyme mimicking activities of HEO, including POD, OXD, and GSHOx.

To evaluate the influence of different gas atmospheres on the HEO/TMB system, the catalytic reaction was separately performed under air, nitrogen (N_2_), and oxygen (O_2_) (Figure S5C, Supporting Information). Under O_2_ (blue line) and air (red line), they display both an obvious absorption peak at A_652_. However, no absorption is observed in N_2_ saturated solution (black line). It highlights the requirement for O_2_ in the catalytic process mediated by HEO. Moreover, the effects of different gas atmospheres on the HEO/TMB/H_2_O_2_ system with increasing time were assessed by recording A_652_ successively every 5 min (Figure S5D, Supporting Information). The curves obtained in O_2_ (blue curve) and air (red curve) exhibit a similar trend with an increase in A_652_ within the first 10 min and a slight decrease in the next 20 min. However, under N_2_ atmospheres, the black curve shows a relatively low A_652_ within the first 20 min compared to the blue and red ones, suggesting the essential role of O_2_ in facilitating the TMB oxidation in the HEO‐catalyzed oxidation process. Further prolonging the reaction time to 30 min leads to a higher A_652_ compared to the values observed under air and O_2_. The results prove that the HEO‐mediated catalytic process is oxygen‐dependent. Furthermore, different catalytic conditions, including of reaction time, pH, and temperature, are optimized in Figure S6 (Supporting Information). Figure S6A,B (Supporting Information) reveals an increase in A_652_ within the first 10 min followed by a decrease from 10 to 30 min. In the presence of HEO, colorless TMB first lost an electron and produced a blue charge‐transfer complex of the parent diamine and diimine with a characteristic A_652_.^[^ ^45^ ^]^ When TMB lost another electron, a yellow quinone‐like diimine yield a characteristic absorption peak at 450 nm, thus leading to the decreased A_652_. Thus, the optimal time was 10 min. As shown in Figure S6C–F (Supporting Information), the optimal pH and temperature were 4.00 and 37 °C, respectively.

The Michaelis–Menten constant (*K*_m_) parameter, reflecting enzymatic efficiency and substrate binding affinity, is a key for characterizing nanozyme performance.^[^ ^46^ ^]^ To determine *K*_m_ and initial maximum reaction velocity (*V*_max_), the catalytic behaviors of HEO with H_2_O_2_ (Figure S7A, Supporting Information) and TMB (Figure S7C, Supporting Information) as substrates were investigated under the optimal conditions, respectively. Their corresponding Lineweaver‐Burk plots are exhibited in Figure S7B,D (Supporting Information). As shown in Figure 2M,N, the *K*_m_ values of HEO for H_2_O_2_ and TMB are 0.27 and 2.40 mm, respectively. It illustrated the preference of HEO to H_2_O_2_ than TMB.^[^ ^47^ ^]^ The affinity of HEO for H_2_O_2_ is significantly higher than that of HRP (3.70 mm).^[^ ^44^ ^]^ The *V*_max_ values for H_2_O_2_ and TMB were measured to be 29.05 × 10^−8^ and 58.79 × 10^−8^ M·s^−1^, respectively, surpassing those reported for HRP (8.71 × 10^−8^ M·s^−1^ for H_2_O_2_ and 10 × 10^−8^ M·s^−1^ for TMB). Moreover, the turnover number (*K*_cat_) of HEO was also evaluated, which represents the rate at which each enzyme molecule (or each active site) converts substrate into product per unit time. The *K*_cat_ can be calculated using the equation *K*_cat_ = *V*_max_/[E] ([E] represents the concentration of HEO). As exhibited in Figure 2M,N, the *K*_cat_ values for H_2_O_2_ and TMB are calculated to be 1.76 × 10^4^ and 3.56 × 10^4^ s^−1^, respectively. They exceed those reported for HRP (3.48 × 10^3^ s^−1^ for H_2_O_2_ and 4 × 10^3^ s^−1^ for TMB),^[^ ^44^ ^]^ suggesting the higher catalytic activity of HEO. The catalytic efficiency of the HEO was further compared with other state‐of‐the‐art nanozymes. Their kinetic parameters including *K*_m_, *V*_max_, and *K*_cat_ are summarized in Figure 2O and Table S1 (Supporting Information). Notably, our HEO achieves a lower *K*_m_ and a higher *V*_max_ of HEO with H_2_O_2_ compared to recently reported high‐entropy nanozymes, including MnFeCoNiCu HEzymes,^[^ ^44^ ^]^ PtPdRuRhIr USHEANPs,^[^ ^48^ ^]^ FeCuAgCeGd‐HEAzyme,^[^ ^49^ ^]^ and AlFeCoCuZn HE‐LDH.^[^ ^50^ ^]^ These results collectively attest to its strong substrate affinity and high intrinsic activity, highlighting the promise of DFT‐guided high‐entropy oxides in nanozyme design.

### 2.3 Catalytic Mechanism of HEO

**Figure 3:**
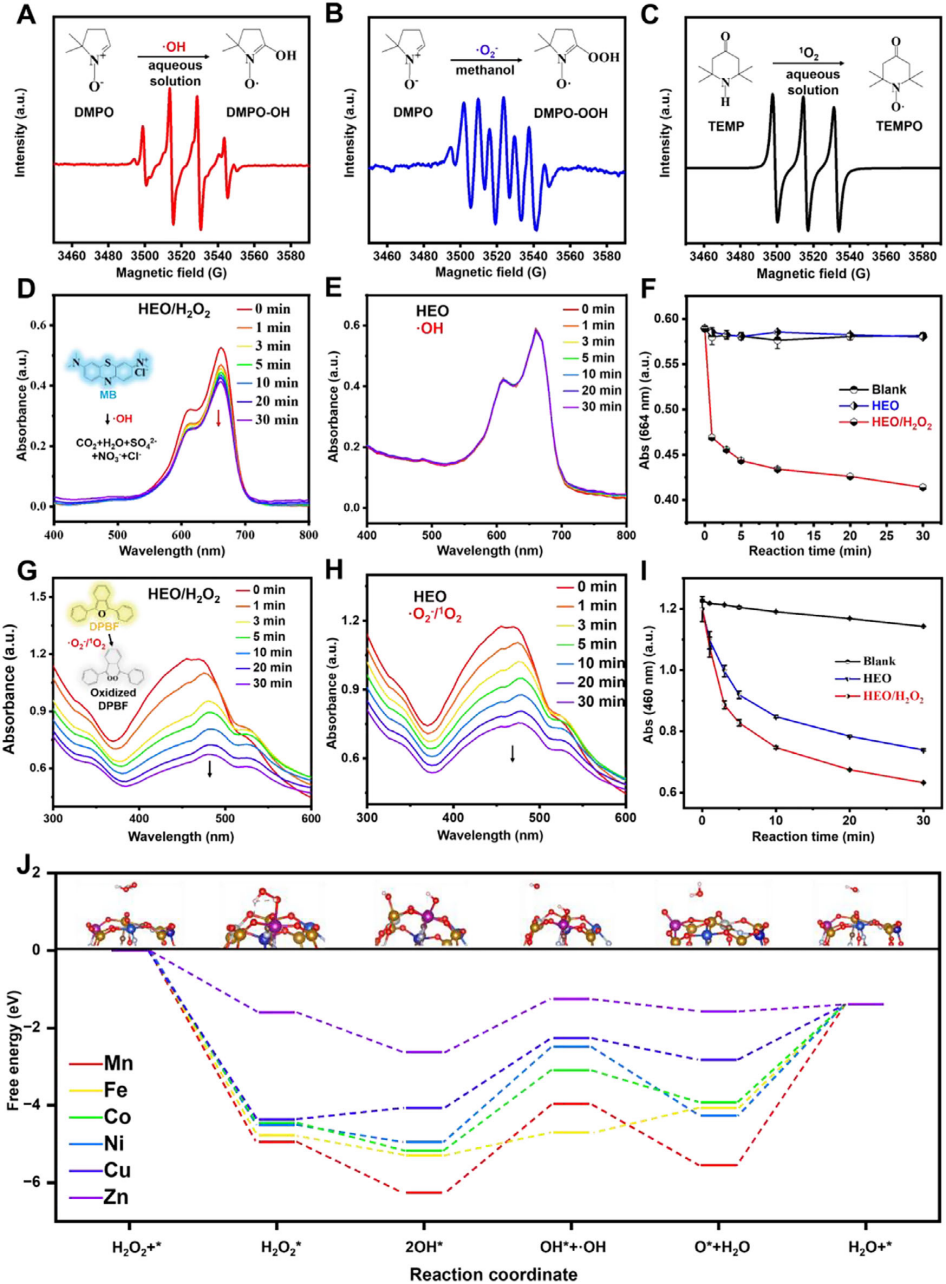
ROS species determination and quantification, together with catalytic mechanisms of HEO by DFT calculations. A) EPR spectra of ·OH, B) ·O_2_^−^, and C) ^1^O_2_ produced from the HEO/TMB/H_2_O_2_ reaction system and corresponding response mechanisms as insets. Time‐dependent changes in UV–vis absorption spectra of MB solutions in D) HEO/H_2_O_2_ and E) HEO systems. F) The change of A_664_ for HEO/MB/H_2_O_2_ (red line), HEO/MB (blue line), and MB (black line) reaction systems within 30 min (*n* = 3). Time‐dependent changes in UV–vis absorption spectra of DPBF solutions in G) HEO/H_2_O_2_ and H) HEO system. I) The change of A_460_ for HEO/DPBF/H_2_O_2_ (red line), HEO/DPBF (blue line), and DPBF reaction systems (black line) within 30 min (*n* = 3). J) The Gibbs free energy profile for the transformation of H_2_O_2_ to H_2_O on individual constituent elements of HEO. The white, red, argent, purple, orange, deep blue, gray, blue, and deep gray atoms represent H, O, N, Mn, Fe, Co, Ni, Cu, and Zn atoms, respectively.

To investigate the mechanism behind the catalytic reaction, the generation of ROS during oxidation was analyzed using electron paramagnetic resonance (EPR) spectroscopy, as shown in **Figure** **3**A–C. Their corresponding response mechanisms are illustrated in the insets. In the HEO/TMB/H_2_O_2_ reaction system, 5,5‐dimethyl‐1‐pyrroline‐N‐oxide (DMPO) can be used as a trapping agent for hydroxyl radical (·OH) in aqueous solution and ·O_2_^−^ in methanol, respectively.^[^ ^57^ ^]^ As depicted by the EPR spectrum in Figure 3A, it presents a secondary characteristic signal of 1:2:2:1.^[^ ^58^ ^]^ Specifically, in an aqueous solution, DMPO acts as a radical scavenger, trapping ·OH to generate DMPO‐OH (Figure 3A inset). The blue line in Figure 3B shows the six‐line signals, which is specific to the ·O_2_^−^. This signal consists of four main peaks and two small peaks, with the intensities of four main peaks being approximately in a 1:1:1:1 ratio.^[^ ^59^ ^]^ It is because the DMPO in a methanol can trap ·O_2_^−^ to form DMPO‐OOH (Figure 3B inset). Besides, 2,2,6,6‐tetramethylpiperidine (TEMP) was used to discriminate singlet oxygen (^1^O_2_).^[^ ^59^ ^]^ A strong 1:1:1 tristate EPR signal is observed after the incubation of HEO with TEMP (Figure 3C), suggesting the generation of ^1^O_2_. The reason is the formation of 2,2,6,6‐tetramethylpiperidine‐1‐oxyl (TEMPO) as depicted in Figure 3C inset. Under acidic conditions, a fraction of ·O_2_^−^ generated in the system could react with H^+^, leading to the production of the ^1^O_2_.^[^ ^60^ ^]^

To quantify the ·OH in the HEO/H_2_O_2_ system, methylene blue (MB) was added (HEO/H_2_O_2_/MB) and reacted for different times between 0 and 30 min. The absorbance spectra are shown in Figure 3D, and the corresponding maximum absorption peak at 664 nm (A_664_) versus time is demonstrated in Figure 3F (red line). For comparison, the HEO/MB (Figure 3E) and MB solutions were also conducted. As depicted in Figure 3F, the A_664_ of the HEO/H_2_O_2_/MB system rapidly declines with increasing time (red line), while the A_664_ of HEO/MB (blue line) and MB (black line) remains almost unchanged. This phenomenon can be attributed to the fact that the ·OH generated from the HEO/H_2_O_2_ system degraded the blue MB into a colorless complex.^[^ ^61^ ^]^ In addition, to compare the amount of ·O_2_^−^ or ^1^O_2_ produced in the HEO/H_2_O_2_ and HEO reaction systems, 1,3‐diphenylisobenzofuran (DPBF) was used with a maximum absorbance at ≈460 nm (A_460_).^[^ ^62^ ^]^ As shown in Figure 3G–I, the A_460_ of both HEO/H_2_O_2_/DPBF (red line) and HEO/DPBF (blue line) systems gradually decrease within 30 min. Moreover, the presence of H_2_O_2_ leads to a more significant drop in the A_460_. The sharp decrease in the A_460_ is attributed to the oxidization of yellow DPBF to a colorless oxidized DPBF by either ·O_2_^−^ or ^1^O_2_.^[^ ^62^ ^]^ In the absence of HEO and H_2_O_2_ (black line in Figure 3I), the A_460_ decreases slightly. These results prove the existence of ROS species involved in the POD‐ and OXD‐like activities for HEO.

To elucidate the electronic structure of HEO in greater depth, the projected DOS (PDOS) for the compositional elements of iron (Fe), zinc (Zn), manganese (Mn), cobalt (Co), nickel (Ni), and copper (Cu) is illustrated in Figure S8 (Supporting Information). Notably, the *d*‐band center of Fe positions closest to the *E*_F_ among these transition metals (Figure S8A, Supporting Information), which facilitated the robust interactions with reaction intermediates, thereby reducing the energy barriers and enhancing catalytic efficiency.^[^ ^63^, ^64^ ^]^ In comparison, the *d*‐band center of Zn is the most distant from the *E*_F_, suggesting that Zn acts as an electron reservoir during the catalytic breakdown of H_2_O_2_ (Figure S8B, Supporting Information), thereby maintaining the valence balance inside the nanozyme. Compared to that of Zn, the *d*‐band centers of Mn, Co, Ni, and Cu situate much closer to the *E*_F_ as shown in Figure S8C–F (Supporting Information), promoting the efficient electron transfer from HEO to H_2_O_2_, further augmenting the overall catalytic performance. Moreover, the *d*‐band centers of Fe, Mn, Co, Ni, and Cu span both occupied and unoccupied states, which not only reduce the energy barrier for electron transfer during the catalytic reaction but also improve the stability of reaction intermediates.^[^ ^65^ ^]^ Guided by DFT‐derived insights into the function of each metal, we systematically synthesized and investigated a series of HEOs with varying molar ratios of the five metal precursors (Mn, Co, Ni, Cu, and Zn), while keeping the total metal concentration constant. As shown in Figure S9 (Supporting Information), the catalytic activity maintains a comparable level when the molar ratio of Zn is reduced from 0.4 to 0.1 (column 2–4), regardless of any change in the molar ratio of Cu. This outcome underscores the primary role of Zn as an electron reservoir, aligns well with the above PDOS analysis of Zn (Figure S8B, Supporting Information). In contrast, decreasing the Cu ratio from 0.4 to 0.1 leads to an obvious decline (column 5 and 6), suggesting that Cu serves as an active site like other metals. It is consistent with the above PDOS analysis of Cu in Figure S8F (Supporting Information). Notably, the highest catalytic performance is achieved when the Zn ratio is lowered while compensating by increasing the proportion of the more catalytically active elements Co and Ni (Mn:Co:Ni:Zn:Cu = 0.4:0.6:0.5:0.1:0.4). In addition, to determine whether expanding the compositional complexity beyond MnCoNiCuZnFe could further enhance enzymatic activity, we extended our study to include Cr‐containing HEOs such as MnCoNiCuCr‐HEO and MnCoNiCuZnCr‐HEO. As shown in Figure S9B (Supporting Information), the addition of Cr to MnCoNiCuZn‐HEO diminished the activity. Conversely, the direct replacement of Zn with Cr leads to an improved catalytic activity, which corroborates the previously discussed PDOS analysis.

Additionally, the Gibbs free energy changes associated with the catalytic decomposition of H_2_O_2_ by HEO were calculated using DFT to elucidate the role of lattice distortion and multi‐metal synergy in lowering the activation barrier.^[^ ^36^, ^66^ ^]^ The lower the binding energy of H_2_O_2_ to metal sites, the higher its adsorption rate.^[^ ^48^ ^]^ As depicted in Figure 3J, the adsorbed H_2_O_2_ molecules are sequentially activated into the H_2_O_2_^*^ intermediate by the Mn, Fe, Ni, Co, Cu, and Zn atoms within the HEO framework.^[^ ^52^ ^]^ Subsequently, the dissociation of H_2_O_2_ on the surface results in the formation of OH^*^ adsorption structure. Following this, one intermediate OH^*^ desorbs from the surface to generate the ·OH, which constitutes the rate‐determining step of the reaction. Among these elements, Fe exhibits the lowest energy requirement for this step (0.59 eV), signifying that Fe serves as the most active site.^[^ ^67^ ^]^ The increased activity can be explained by the higher concentration of electronic states near the *E*_F_ for Fe (Figure S8A, Supporting Information), which imparts greater reactivity and metallic character to this element. Meanwhile, other elements including Mn, Co, Ni, and Cu further amplify the catalytic performance through synergistic interactions, thereby contributing to the overall optimization of functional properties. Subsequently, the adsorbed OH^*^ undergoes further dissociation, releasing a proton (H^+^) to form an adsorbed oxygen species (O^*^) and a water molecule (H_2_O).^[^ ^68^ ^]^ Finally, as the O^*^ intermediate experiences two consecutive hydrogenation steps followed by desorption to form H_2_O molecules, the active site reverts to its initial state, completing the catalytic cycle.^[^ ^69^ ^]^ In conclusion, DFT calculations offer comprehensive insights into the electronic structure and catalytic mechanism of the HEO, while also reveal the synergistic effects among its transition metal elements that support the POD‐like activity.

### 2.4 Colorimetric Detection of GSH

**Figure 4:**
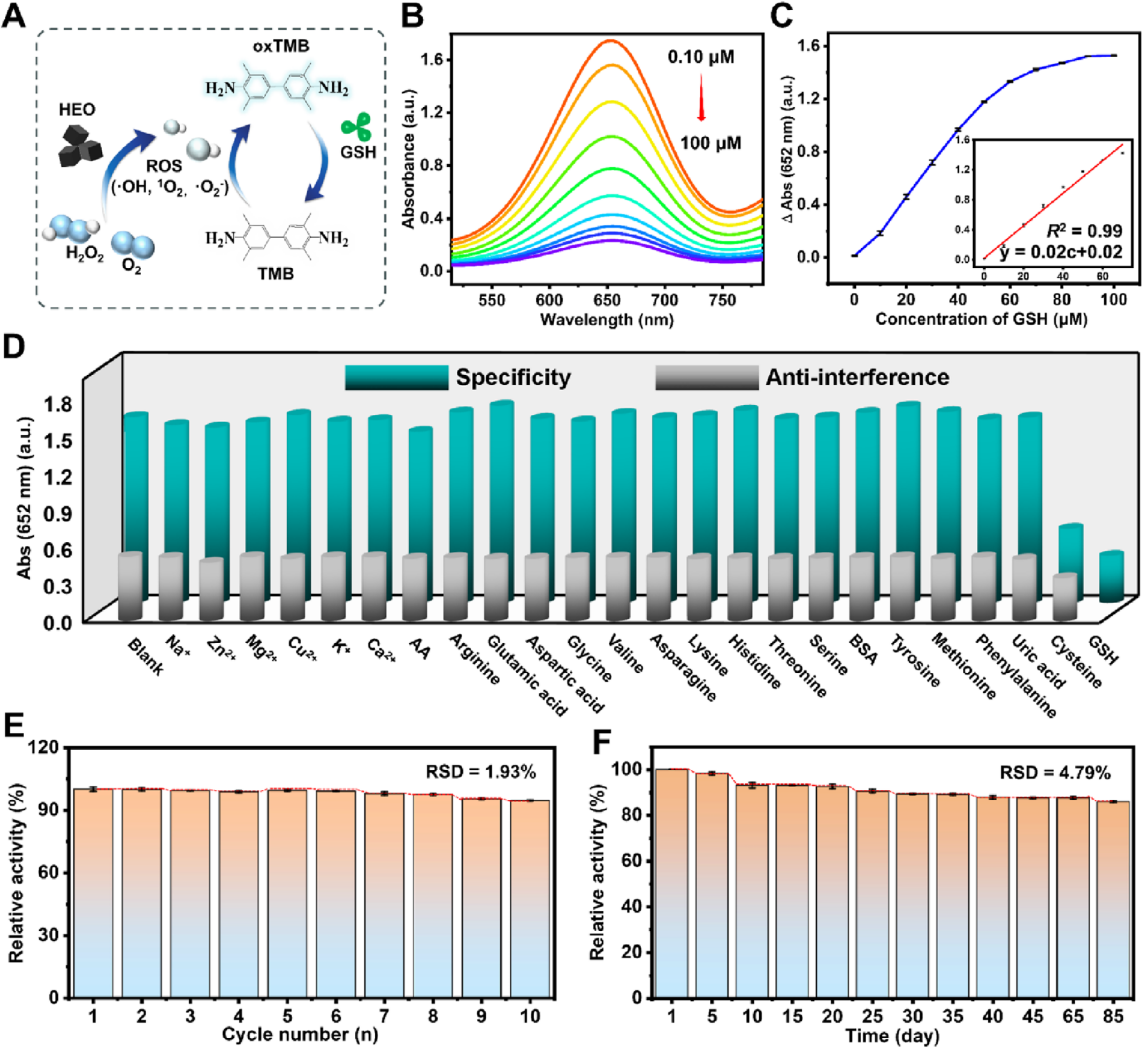
Sensitivity, selectivity, and stability for the HEO/TMB/H_2_O_2_ based colorimetric system. A) Schematic diagram of the HEO/TMB/H_2_O_2_ reaction system for the detection of GSH. B) UV–vis absorbance spectra of HEO/TMB/H_2_O_2_ system in the presence of GSH concentrations between 0.10 and 100 µm. C) Corresponding relationship between ΔA_652_ and GSH concentrations (*n* = 3). Inset is the fitting curve. D) Specificity (cyan column) and anti‐interference (gray column) tests for the HEO/TMB/H_2_O_2_ system. Note: GSH and Cys, 50 µm; AA, 0.20 mm, other substances (Na^+^, Zn^2+^, Mg^2+^, Cu^2+^, K^+^, Ca^2+^, Arg, Glu, Asp, Gly, Val, Asn, Lys, His, Thr, Ser, BSA, Tyr, Met, Phe, and uric acid), 2 mm. E) Cycling stability of HEO through 10 successive catalytic cycles monitored by HEO/TMB/H_2_O_2_ system (recorded by the relative enzyme activity) (*n* = 3). F) Storage stability of HEO up to 85 days, recorded by the relative enzyme activity. Error bars represent the standard deviations of three independent experiments (*n* = 3).

The addition of GSH into the above HEO/TMB/H_2_O_2_ system causes the fading of the blue color and thus leads to the reduction of the A_652_ (**Figure** **4**A). Figure 4B illustrates the decreased absorbance spectra with increasing GSH concentrations from 0.10 to 100 µm, and the corresponding calibration curve is shown in Figure 4C. The HEO/TMB/H_2_O_2_ system provides a linear response range between 0.10 and 70 µm (Figure 4C inset) and a low limit of detection (LOD) of 71.22 nm. It is comparable to the previously reported nanozymes listed in Table S2 (Supporting Information). In addition to the POD‐like activity of HEO, we also employed the GSHOx‐like activity for GSH detection. Different concentrations of GSH (1 µm‐1 mm) were added to an HEO/DTNB‐containing PBS buffer (pH 6.50). The absorbance spectra and corresponding relationship between A_412_ and GSH concentrations are displayed in Figure S10 (Supporting Information). An obvious increase in A_412_ along with the rising GSH concentrations from 1 to 300 µm is observed in Figure S10B (Supporting Information). The HEO/DTNB catalytic system exhibits a linear response range of 1–224 µm and a low LOD of 0.28 µm. Thus, the prepared HEO‐based systems present an efficient platform for GSH determination.

The specificity and anti‐interference are key parameters to evaluate the efficiency of HEO for GSH detection. Various interferents including Na^+^, Zn^2+^, Mg^2+^, Cu^2+^, K^+^, Ca^2+^, ascorbic acid (AA), arginine (Arg), glutamic acid (Glu), aspartic acid (Asp), glycine (Gly), valine (Val), asparagine (Asn), lysine (Lys), histidine (His), threonine (Thr), serine (Ser), bovine albumin (BSA), tyrosine (Tyr), methionine (Met), phenylalanine (Phe), uric acid, and cysteine (Cys) were used to test the selectivity. Figure 4D (cyan column) shows that only 50 µm GSH and Cys decrease the A_652_ remarkably, while other substances have nearly no effect on A_652_. As shown in Figure S11A, only the presence of GSH and Cys caused obvious fading in the reaction solutions. Similarly, the anti‐interference test in Figure 4D (gray column) exhibits that the coexistence of Cys with GSH has a slight decrease in A_652_. It is well‐known that the concentration of Cys (µm level) in the human body is much lower than that of GSH (mm level).^[^ ^70^ ^]^ Therefore, the presence of Cys would not interfere with the GSH detection in physiological fluids. Moreover, a remarkably high AA concentration of 0.20 mm (at least 20‐fold above the physiological level)^[^ ^70^ ^]^ does not induce any significant interference in GSH quantification, suggesting the robustness of HEO/TMB/H_2_O_2_ system. The results confirm the high selectivity and anti‐interference in GSH detection. Additionally, the cycling and storage stability of HEO were systematically evaluated, respectively. As can been seen form Figure 4E, our HEO retains its initial activity above 95% after 10 cycles, with a relative standard deviation (RSD) of 1.93%, demonstrating its exceptional operational robustness. When stored at 4 °C for 85 days, it preserves above 86% of its initial activity, with a RSD of 4.79%, underscoring its superior long‐term stability (Figure 4F). These results collectively highlight the outstanding reusability and long‐term performance of HEO, making it highly suitable for practical applications.

To assess the biocompatibility of the HEO nanozymes for potential biomedical applications, we evaluated their cytotoxicity against HEK293 cells using the MTT assay. Different concentrations of the HEO within 0–250 µg·mL^−1^ were incubated with cells for 6 h, respectively. The results in Figure S12 (Supporting Information) revealed that the cell viability maintained above 89% even at the highest concentration of 250 µg·mL^−1^, indicating low cytotoxicity and good biocompatibility.

To validate the GSH determination in real samples, the goat serum was utilized based on the standard addition method. Besides, HPLC was also used to detect GSH and evaluate the accuracy of the HEO/TMB/H_2_O_2_ system. The HPLC chromatogram of standard GSH in Figure S13A (Supporting Information) shows a sharp peak at 3.58 min. A linear calibration curve (R^2^ > 0.99) is established based on peak areas (Figure S13B, Supporting Information). The obtained recovery rates range from 98.30% to 102.57%, as detailed in Table S3 (Supporting Information). Besides, the results showcase that the HEO‐based colorimetric method aligns well with the HPLC results. It proves the accuracy of the colorimetric HEO/TMB/H_2_O_2_ system in real applications.

**Figure 5:**
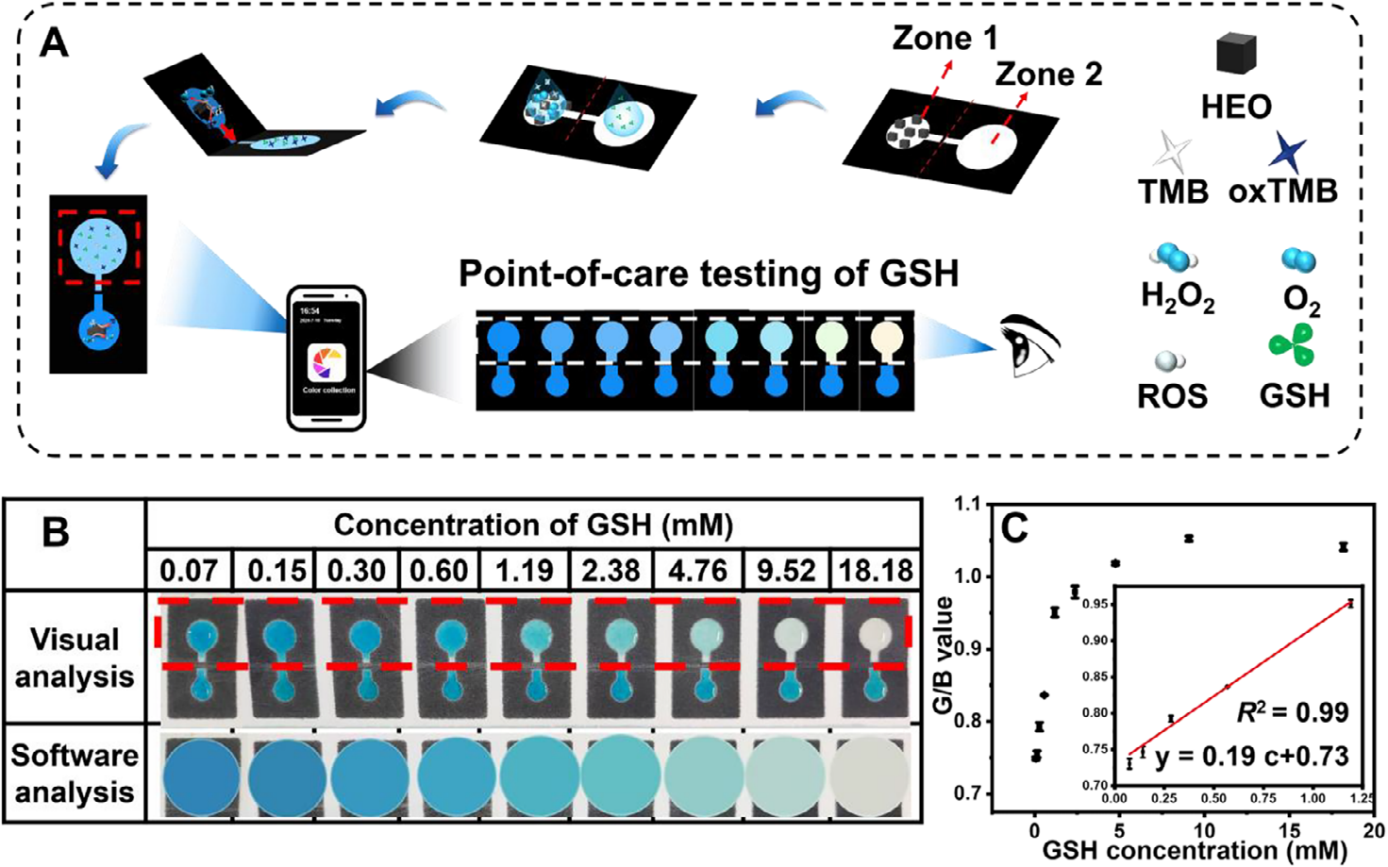
On‐site GSH detection via a portable µPAD in the aid of smartphone RGB analysis. A) Illustration of smartphone‐assisted µPAD for the colorimetric detection of GSH. B) Photos of the HEO/TMB/H_2_O_2_ colorimetric system with different GSH concentrations between 0.07 and 18.18 mm and corresponding color change obtained from the software analysis. C) The relationship of G/B values with different GSH concentrations. Inset is the fitting curve between the G/B values and GSH concentrations (*n* = 3).

The µPAD enjoys the advantages of simple operation, low cost, and portability. In particular, the µPAD is created by wax printing on a single sheet of chromatographic paper, with the pattern design shown in Figure S1 (Supporting Information). The black section is a hydrophobic barrier, which is made of wax. As illustrated in **Figure** **5**A, zone 1 and 2 serves as the reaction and detection sections, respectively. It facilitates the fabrication of a ready‐to‐use dumbbell‐type µPAD. The mixed solution containing TMB and H_2_O_2_ is first added to zone 1 with HEO for 5 min and then flows to zone 2 with GSH through folding. The color change of zone 2 is captured and analyzed by a smartphone app with red (R), green (G), and blue (B) color space, allowing portable detection of GSH. As shown in Figure 5B, the color in zone 2 (red line) gradually fades along with the increasing GSH concentrations from 0.07 to 18.18 mm. Besides, it presents a positive correlation of green and blue color intensity ratio (G/B value) with GSH concentrations of 0.07–9.52 mm (Figure 5C). Based on the curve in Figure 5C, a linear range of 0.07–1.73 mm and a LOD of 32.67 µm is obtained (inset of Figure 5C). As a critical biomarker for various pathological conditions, extracellular GSH plays an important role in detoxification processes and protection against oxidant injury.^[^ ^71^ ^]^ The physiological concentration of extracellular GSH is in the micromolar range, for example, 0.002 mm for human plasma and 1.37 mm for human blood.^[^ ^72^, ^73^ ^]^ Thus, the sensitivity of our µPAD assay is sufficient for monitoring extracellular GSH levels. To study the potential influence of light conditions on the RGB method, three different lighting conditions, including natural light, filament lamp (original experiments), and dark environment, were employed. As shown in Figure S14A (Supporting Information), the color transitions of the reaction solutions exhibit clear and consistent changes with increasing GSH concentrations from 0 to 0.8 mm. Furthermore, quantitative analysis of the G/B values derived from these images (Figure S14B, Supporting Information) reveals a strong positive correlation with GSH concentration across all lighting settings. Importantly, these results demonstrate that the G/B readout is nearly unaffected by ambient light, confirming the robustness of our method under varying illumination conditions. This simple and effective HEO‐based POCT platform underscores its potential for practical and on‐site GSH detection.

## 3 Conclusion

In summary, a series of PBAs with varying metal compositions, including binary (ZnFe), ternary (CuZnFe), quaternary (NiCuZnFe), quinary (CoNiCuZnFe), and high‐entropy (MnCoNiCuZnFe) were initially investigated using DFT calculations. Experimental results confirmed that the HEO possessed the highest catalytic activity, corroborating the accuracy of DFT calculations. Compared to the simplified PBA models lacking cyanide bridges, the TDOS revealed a notable increase in the *d*‐band centers across all ZnFe, CuZnFe, NiCuZnFe, CoNiCuZnFe, and MnCoNiCuZnFe nanozymes. This suggests that cyanide groups play a crucial role in modulating the electronic properties of the metal centers in PBAs, facilitating efficient electron delocalization along the metal‐cyanide‐metal pathway and thereby influencing catalytic performance. The molar ratios for the five metal precursors were optimized at 0.4:0.6:0.5:0.1:0.4 for Mn:Co:Ni:Zn:Cu, yielding the highest catalytic activity. Under optimal conditions, the proposed HEO/TMB/H_2_O_2_ based colorimetric system exhibited an exceptionally low detection limit of 71.22 nm for GSH within the linear range of 0.10–70 µm. The system also demonstrated outstanding selectivity, showing strong anti‐interference capability against 23 potential interferents. Besides, it enjoyed remarkable cycling (at least 10 cycles) and storage stability (up to 85 days) together with remarkable cell viability. Moreover, the HEO demonstrated satisfactory reliability and accuracy in the recovery of GSH in diluted goat serum. DFT calculations systematically elucidated the Gibbs free energy of H_2_O_2_ dissociation on individual metal sites including Mn, Co, Ni, Cu, Zn, and Fe, manifesting the multi‐element synergistic effects in high‐entropy materials. To achieve POCT, a µPAD‐based colorimetric platform was developed for rapid, convenient, and sensitive GSH monitoring, featuring a broad detection range (0.07–1.73 mm) and a low detection limit (32.67 µm). It was demonstrated that different lighting conditions caused negligible influence on the GBR colorimetric method. The innovative design underscores the remarkable sensitivity and practicality for bioanalytical applications. These findings not only advance the understanding of better catalytic activity of HEO than low‐ and medium‐entropy metal oxides, but also establish a versatile platform for biosensing and environmental monitoring.

## 4 Experimental Section

### Materials and Instruments

Potassium ferricyanide, sodium citrate, cobalt nitrate hexahydrate, cupric nitrate trihydrate, manganese nitrate tetrahydrate, nickel nitrate hexahydrate, zinc nitrate hexahydrate, chromium(III) nitrate, sodium acetate (CH_3_COONa, NaAc), dimethyl sulfoxide, 3,3′,5,5′‐tetramethylbenzidine (TMB), 1,3‐diphenylisobenzofuran (DPBF), methylene blue trihydrate (MB), glutathione (GSH), acetic acid (CH_3_COOH, HAc), hydrogen peroxide (H_2_O_2_, 30 wt.%), polyethylene glycol (PEG, Mw = 2000), 5,5′‐dithiobis‐(2‐nitrobenzoic acid) (DTNB), 5,5‐dimethyl‐1‐pyrroline‐N‐oxide (DMPO), 2,2,6,6‐tetramethylpiperidine (TEMP), bovine serum albumin (BSA), ascorbic acid (AA), and various salts were purchased from Sigma–Aldrich (America). Arginine, glutamic acid, aspartic acid, glycine, valine, asparagine, lysine, histidine, threonine, serine, bovine albumin, tyrosine, methionine, phenylalanine, uric acid, and cysteine were purchased from Sinopharm Chemical Reagent (China). All reagents used in this study were of analytical grade and used without further purification. Ultrapure water (18.25 MΩ·cm) prepared using a PURIST UV ultrapure water system (RephiLe Bioscience, China) was used in the experiments.

The morphology of nanomaterials was observed by transmission electron microscopy (TEM, JEM‐2100, JEOL, Japan, operated at 200 kV) and atomic force microscopy (AFM, Multimode 8‐HR, Bruker, Germany) X‐ray diffraction spectrum (XRD) was used to study the phase and crystal structure with a Bruker D8 advanced X‐ray diffractometer (Smart Lab 9 KW, Rigaku, Japan). The chemical components were investigated by X‐ray photoelectron spectrometer (ESCALAB 250Xi, Thermo Fisher Scientific, America). To capture the reactive oxygen species (ROS) generated in the catalytic system, electron paramagnetic resonance (EPR) measurements were conducted on a Bruker EMX‐8/2.7 electron paramagnetic resonance spectrometer. The adsorption data was obtained using a high‐performance surface area and aperture analyzer (ASAP 2460, Micromeritics, America). Based on the adsorption data, the specific surface area, pore size, and pore volume were calculated by the Bruauer–Emmet‐Teller (BET) and Barrett–Joyner–Halenda (BJH) methods. The true density of HEO was measured using a fully automated gas pycnometer (Micromeritics AccuPyc II 1345, America). The UV–vis absorbance spectra were measured on a U‐5100 spectrophotometer (Hitachi, Japan). The GSH concentrations were determined by the 1260 Infinity high‐performance liquid chromatography (HPLC, Agilent technologies, America) method. Error bars represent the standard deviation (SD) derived from at least three independent replicates (*n* = 3).

### Synthesis of PBA Oxides

For the preparation of high entropy Prussian blue analogue (PBA), in a typical procedure,^[^ ^19^ ^]^ 0.40 mmol manganese nitrate tetrahydrate, 0.40 mmol cobalt nitrate hexahydrate, 0.40 mmol nickel nitrate hexahydrate, 0.40 mmol cupric nitrate trihydrate, 0.40 mmol zinc nitrate hexahydrate, and sodium citrate (2.25 mmol) were dissolved in 50 mL of ultrapure water to prepare a clear solution A. In a separate step, 2 mmol of K_3_[Fe(CN)_6_] was dissolved in 50 mL of ultrapure water to form a clear solution B. Solution B was then gradually added to solution A while stirring at room temperature. After 10 min of continuous stirring, the resulting mixture was left to age at room temperature for 24 h. Afterward, the product was collected by centrifugation and washed repeatedly with ultrapure water and ethanol. The obtained powder was calcined in a muffle furnace at 150 °C for 1 h, producing the final high‐entropy oxide (HEO, MnCoNiCuZnFe). The synthesis steps for binary, ternary, quaternary, and quinary PBA oxides were similar to that of HEO, except for the substitution of the composition of metal nitrates in solution A. To prepare binary PBA oxide, 2 mmol of zinc nitrate hexahydrate was added to solution A. To prepare ternary PBA oxide, the solution A contained 1 mmol of zinc nitrate hexahydrate and 1 mmol of cupric nitrate trihydrate. To prepare quaternary PBA oxide, 0.66 mmol of zinc nitrate hexahydrate, 0.67 mmol of cupric nitrate trihydrate, and 0.67 mmol of nickel nitrate hexahydrate were added to solution A. To prepare quinary PBA oxide, 0.50 mmol of zinc nitrate hexahydrate, 0.50 mmol of cupric nitrate trihydrate, 0.50 mmol of nickel nitrate hexahydrate, and 0.50 mmol of cobalt nitrate hexahydrate made up solution A. Besides, the effect of different molar ratios among the five metal precursors (Mn:Co:Ni:Zn:Cu) in the HEO was also examined while keeping the total metal concentration constant (2.0 mm). Moreover, to study the influence of Cr on the activity, Cr‐containing HEOs were also prepared, namely MnCoNiCuCr‐HEO and MnCoNiCuZnCr‐HEO. They were prepared with all metals in an equimolar ratio, while maintaining a constant total metal concentration of 2.0 mm.

### Peroxisase‐Like Activity

In order to optimize the catalytic conditions, HEO (25 µg·mL^−1^), TMB (0.15 mm), and H_2_O_2_ (2.5 mm) system (HEO/TMB/H_2_O_2_) was reacted separately for different times (5, 10, 15, 20, 25, and 30 min), with different pH values (3.60, 4.00, 4.50, 5.00, and 5.80 in 0.20 mm NaAc‐HAc buffer), and at different temperatures (20, 30, 37, 45, and 50 °C). The corresponding absorption spectra were recorded by UV–vis spectrophotometer and the maximum absorbance at 652 nm (A_652_) was obtained. In order to study the steady‐state kinetic analysis, the A_652_ of the reaction system was recorded with various concentrations of TMB (0.10 to 0.90 mm) and H_2_O_2_ (0.90 to 9 mm) in NaAc‐HAc buffer (0.20 mm at pH 4.00) for 10 min at 37 °C, respectively. Then, the A_652_ was converted to the concentration of oxidized TMB (oxTMB) according to Lambert–Beer law (the molar absorption coefficient of TMB is 3, 9000 mol L^−1^ cm^−1^). The error bars in all cases indicate the standard deviation derived from a minimum of three measurements. The kinetic parameters, including the maximum reaction rate (*V*max, 10^−8^ M s^−1^) and the Michaelis constant (*K*m, mm), of the nanozymes were derived by fitting the experimental data to the Michaelis–Menten Equation (1), where [S] is the substrate (TMB or H_2_O_2_) concentration.

(1) $$V=\frac{V_{\max}\left[S\right]}{K_{m}+[S]}$$

### Oxidase and Glutathione Oxidase‐Like Activity

To identify the oxidase‐like activity of HEO, a buffer solution of NaAc‐HAc containing HEO (25 µg·mL^−1^) and TMB (0.15 mm) was reacted at room temperature for 5 min. Similarly, the glutathione oxidase‐like activity was investigated by adding GSH (0.15 mm) and HEO (150 µg·mL^−1^) to PBS buffer (pH 6.50) for 5 min in the dark. Then, the above reaction solution was filtered by 0.22 µm filter to obtain a clear and transparent solution. After the addition of DTNB (0.10 mm) for 5 min, the UV–vis absorption spectra were obtained. The reaction between colorless DTNB and GSH produces 2‐nitro‐5‐thiobenzoic acid, a yellow compound with a strong absorption peak at ≈412 nm.

In order to investigate the response performance of HEO toward GSH, a series of GSH solutions with concentrations ranging from 1 µm to 1 mm were mixed with HEO (150 µg·mL^−1^) and DTNB (0.10 mm) in PBS buffer (pH 6.50). The UV–vis absorption spectra of the mixture solutions were recorded after incubation in the dark for 5 min and followed by filtration with a 0.22 µm membrane filter.

### Radical Species

In order to detect hydroxyl radical (·OH), DMPO, serving as a trapping agent, was introduced into a mixed NaAc‐HAc buffer solution containing HEO (0.50 mg·mL^−1^) and H_2_O_2_ (500 mm). To detect superoxide radical anions (·O^2−^), DMPO in methanol was added to the HEO/H_2_O_2_ mixed solution. To detect singlet oxygen (^1^O_2_), TEMP was added to the HEO/H_2_O_2_ mixed solution. In order to quantify the ·OH produced by the reaction, HEO (50 µg·mL^−1^) and MB (7.50 µm) were added to NaAc‐HAc buffer with or without H_2_O_2_ (10 mm) for different reaction times. The absorption spectra were recorded and the maximum absorbance at 664 nm were obtained. To measure the levels of ^1^O_2_/·O^2−^ generated during the reaction, a mixture of HEO (50 µg·mL^−1^) and DPBF (50 µg·mL^−1^) was introduced into NaAc‐HAc buffer with or without H_2_O_2_ (10 mm). The UV–vis absorption profiles of the catalytic system were measured, and the peak absorption value at ≈460 nm was determined.

### Density Functional Theory (DFT) Calculation

All computational investigations based on DFT were executed using the Vienna Ab initio Simulation Package (VASP).^[^ ^74^ ^]^ The electronic exchange‐correlation interactions were approximated through the generalized gradient approach (GGA) with the Perdew–Burke–Ernzerhof (PBE) functional.^[^ ^75^ ^]^ The ionic cores were represented by the projected augmented wave (PAW) formalism, which taking valence electrons into account using a plane wave basis set with a kinetic energy cutoff of 450 eV.^[^ ^76^ ^]^ The convergence threshold for solving the Kohn–Sham equations iteratively was established at 10^−5^ eV. Structural optimizations were considered complete when the forces acting on each atom decreased below 0.02 eV·Å^−1^. Sampling of the Brillouin zones was performed using a Gamma‐centered *k*‐point grid of dimensions 3 × 3 × 3.^[^ ^77^ ^]^ Our DFT calculations were performed in the vacuum according to the former literatures.^[^ ^29^, ^44^, ^60^ ^]^ The main goal is to identify trends in activity across different PBA nanozymes and establish the fundamental reaction mechanisms. Since the trends in electronic structure and the qualitative features of the reaction pathways are generally consistent between vacuum and solvated environments,^[^ ^78^, ^79^, ^80^ ^]^ our key conclusions regarding the relative performance of the nanozymes remain valid.

### Sensitivity, Selectivity, and Stability

To explore the sensitivity for GSH determination, a series of GSH concentrations from 0.10 to 100 µm were added to the HEO/TMB/H_2_O_2_ reaction system. After reaction for 10 min at 37 °C, the absorption spectra were recorded. To assess the specificity, potential interferences from compounds including arginine, glutamic acid, aspartic acid, glycine, valine, asparagine, lysine, histidine, threonine, serine, bovine albumin, tyrosine, methionine, phenylalanine, uric acid, cysteine, Ca^2+^, K^+^, Cu^2+^, Mg^2+^, Zn^2+^, Na^+^, and AA were systematically analyzed. Moreover, to assess the anti‐interference analysis, the interfering substances were added to the HEO/TMB/H_2_O_2_ reaction system containing GSH to detect the A_652_. To evaluate the cycling stability, the HEO (1 mg mL^−1^) was collected by centrifugation, washed with buffer solutions and redispersed in fresh TMB/H_2_O_2_ reaction solution after each reaction cycle. Besides, to study the stability of HEO, the peroxidase‐like activity of the reaction system was performed periodically within 85 days.

### Cytotoxicity Assays

Human embryonic kidney (HEK293) cells obtained from the Institute of Biochemistry and Cell Biology, Chinese Academy of Sciences (Shanghai, China), were cultured in DMEM high‐glucose medium supplemented with 10% (v/v) FBS and 1% (v/v) antibiotics (penicillin/streptomycin). Cell cultures were maintained at 37 °C in a humidified incubator with 5% CO_2_. Cytotoxicity assays of HEO were performed using MTT according to the manufacturer's instructions. Briefly, HEK293 cells (≈1 × 10^4^/well) were seeded in 96‐well plates and cultured in cell medium for 24 h. Different concentrations of HEO ranging from 0 to 250 µg·mL^−1^ were then incubated with HEK293 cells for 24 h at 37 °C. After incubation, the culture medium was removed, and each well was washed with PBS solution. MTT (0.5 mg mL^−1^) were added to each well, followed by a 4 h incubation. After the supernatant was removed, 120 µL of dimethyl sulfoxide was added to each well, and the plate was shaken for 10 min to dissolve the methyl crystal. The absorbance values at 490 nm (OD_490nm_) for each well were recorded using a microplate reader (Synergy 2, BioTek).

### Recovery of GSH in Diluted Goat Serum

To assess the practicability, goat serum samples (diluted 10‐fold) mixed with varying GSH concentrations, including 5, 10, 20, and 30 µm was added into the HEO/TMB/H_2_O_2_ reaction system for 10 min at 37 °C to obtain A_652_. For comparison, HPLC was also utilized to detect the samples. Chromatographic separation was conducted on a C18 column using an isocratic mobile phase consisting of 50 mm potassium dihydrogen phosphate (adjusted to pH 2.50 with phosphoric acid) and methanol (90: 10, v/v) at a flow rate of 0.60 mL min^−1^. The column temperature was maintained at 30 °C, and analytes were monitored at 210 nm by UV detection.

### GSH Detection by µPAD

In order to realize the point‐of‐care testing (POCT) of GSH, a microfluidic paper‐based analytical device (µPAD) with an origami design involved wax printing to create hydrophobic barriers on paper, was employed for the HEO‐based system. The schematic design of the µPAD is depicted in Figure S1 (Supporting Information). To immobilize the HEO on the µPAD surface, 20 µL of the HEO solution (0.20 mg·mL^−1^) was drop‐casted in zone 1 and dried at 50 °C. Simultaneously, zone 2 was treated with PEG (5 mm). Following air drying, the fully assembled µPAD were ready for use. After slowly folded in half, the solution in zone 1 flowed to zone 2 with 5 µL of GSH solution to initiate the colorimetric reaction. The generated colorimetric signals were captured as JPEG images with a smartphone RGB analysis software (Fuzhou Wanxiang Secai Technology Co., Ltd., China). The obtained images were processed by separating the color into the red, green and blue channels. The green and blue channels were used to quantify the colorimetric change of paper devices. To evaluate detection limits, 5 µL of various concentrations of GSH were added to Zone 2 to obtain the final concentration of 0–18.18 mm and assays were performed as described above.

To investigate the potential effect of light on the RGB method, similar experiments were carried out at three different lighting conditions including natural light, filament lamp (above experiments), and complete darkness. Specially, 30 µL of the HEO solution (0.20 mg·mL^−1^) and 10 µL of different concentrations of GSH solutions was drop‐casted in zone 1 and 2, respectively. After complete reaction, colorimetric signals were captured and analyzed by RGB analysis software.

## Conflict of Interest

The authors declare no conflict of interest.

## Supporting information



Supporting Information

## Data Availability

The data that support the findings of this study are available from the corresponding author upon reasonable request.
