# Supplementary material for: DFT‐Guided Design of a Multi‐Enzyme Mimetic High‐Entropy Nanozyme for Cascaded Glutathione Detection and Point‐of‐Care Testing
Source: Adv Sci (Weinh). 2025 Nov 5;13(4):e15765. doi: 10.1002/advs.202515765 (PMC12822477; doi:10.1002/advs.202515765)
Supplement: Supplementary file 1 — Supporting Information [file ADVS-13-e15765-s001.docx]

Supporting Information

**DFT-Guided Design of a Multi-Enzyme Mimetic High-Entropy Nanozyme for Cascaded Glutathione Detection and Point-of-Care Testing**

*Hua Lin^1^, Li Ke^1^, Shuran Wang^1^, Ruoke Li^1^, Shengmin Zhou^1^, Yueling Liu*^1^, Huan Pang*^2^*

1 State Key Laboratory of Bioreactor Engineering, School of Biotechnology, East China University of Science and Technology, Shanghai 200237, P. R. China

2 School of Chemistry and Chemical Engineering, Yangzhou University, Jiangsu 225009, P. R. China

*Corresponding author.

Email: yuelingliu@ecust.edu.cn (Y. L.); panghuan@yzu.edu.cn (H. P.).

Fax: +86-21-6425-2257;

Tel: +86-21-6425-2257.


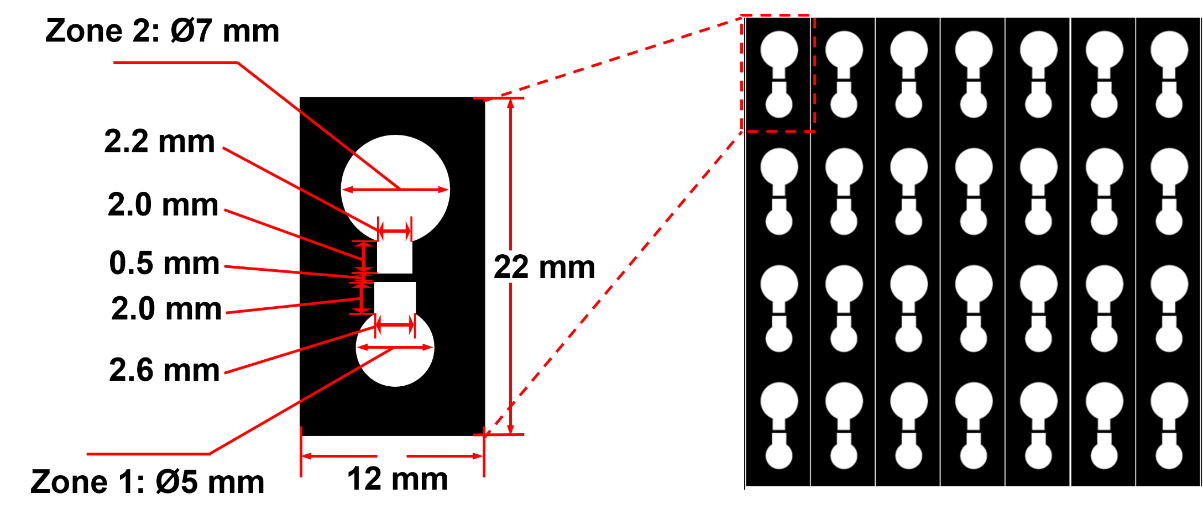


**Figure S1.** The design and dimensions of the μPAD on a single sheet of chromatography paper.


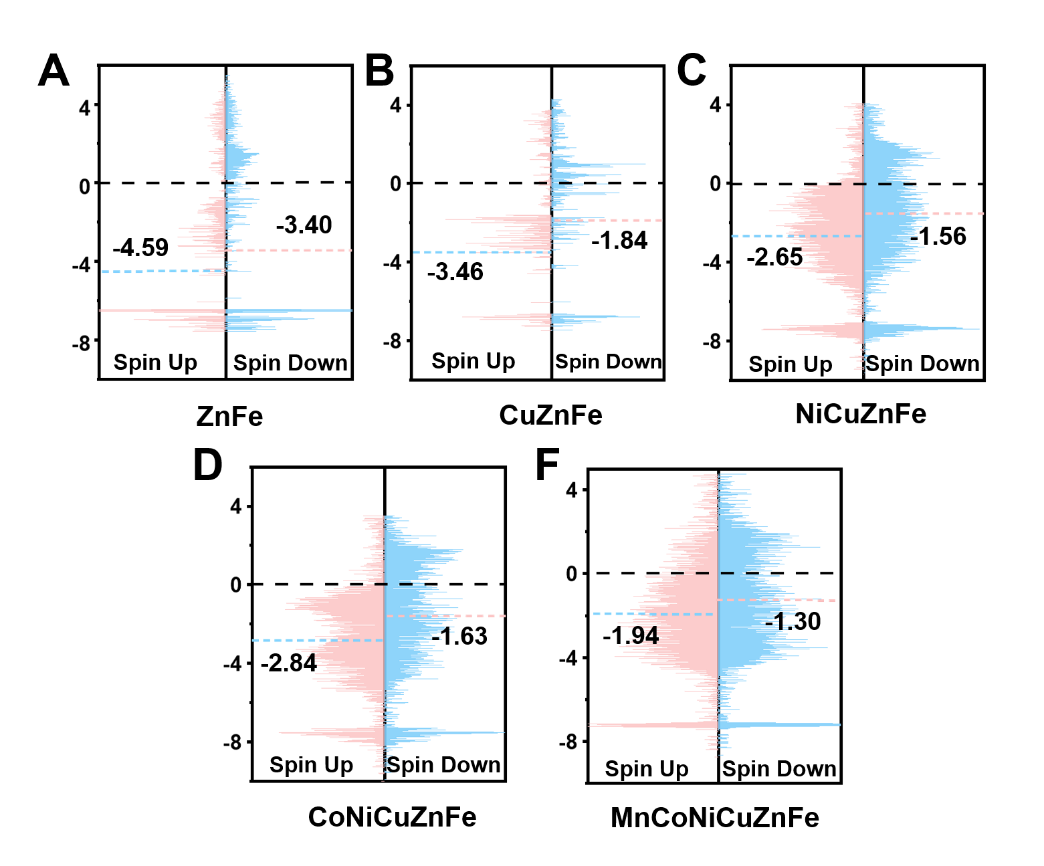


**Figure S2.** TDOS for A) ZnFe, B) CuZnFe, C) NiCuZnFe, D) CoNiCuZnFe, and E) MnCoNiCuZnFe based nanozymes without cyanide bridges (simplified PBA models).


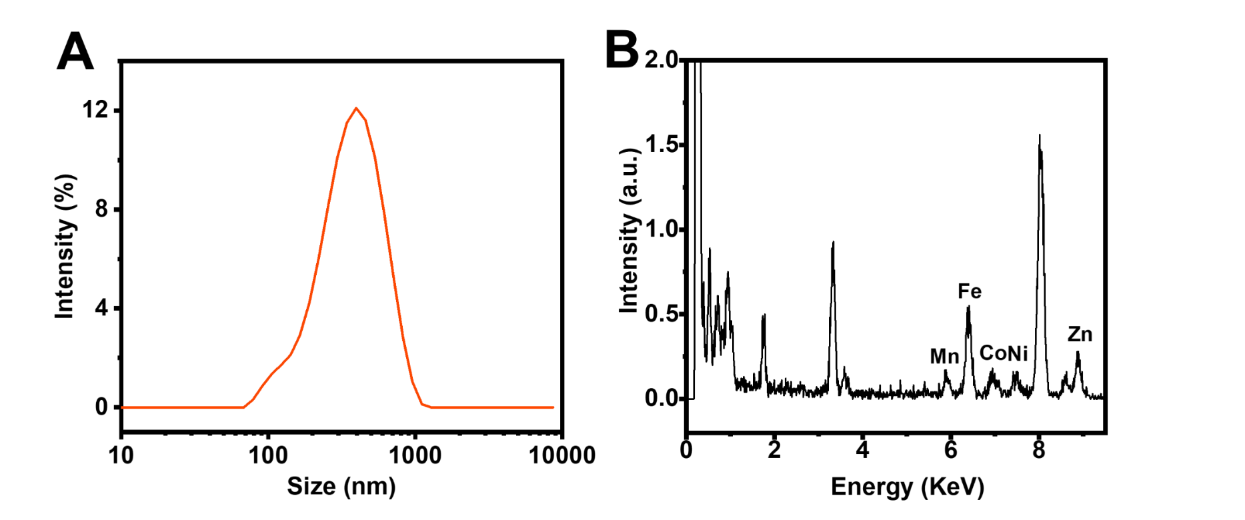


**Figure S3.** A) DLS and B) EDS spectra of HEO (MnCoNiCuZnFe).


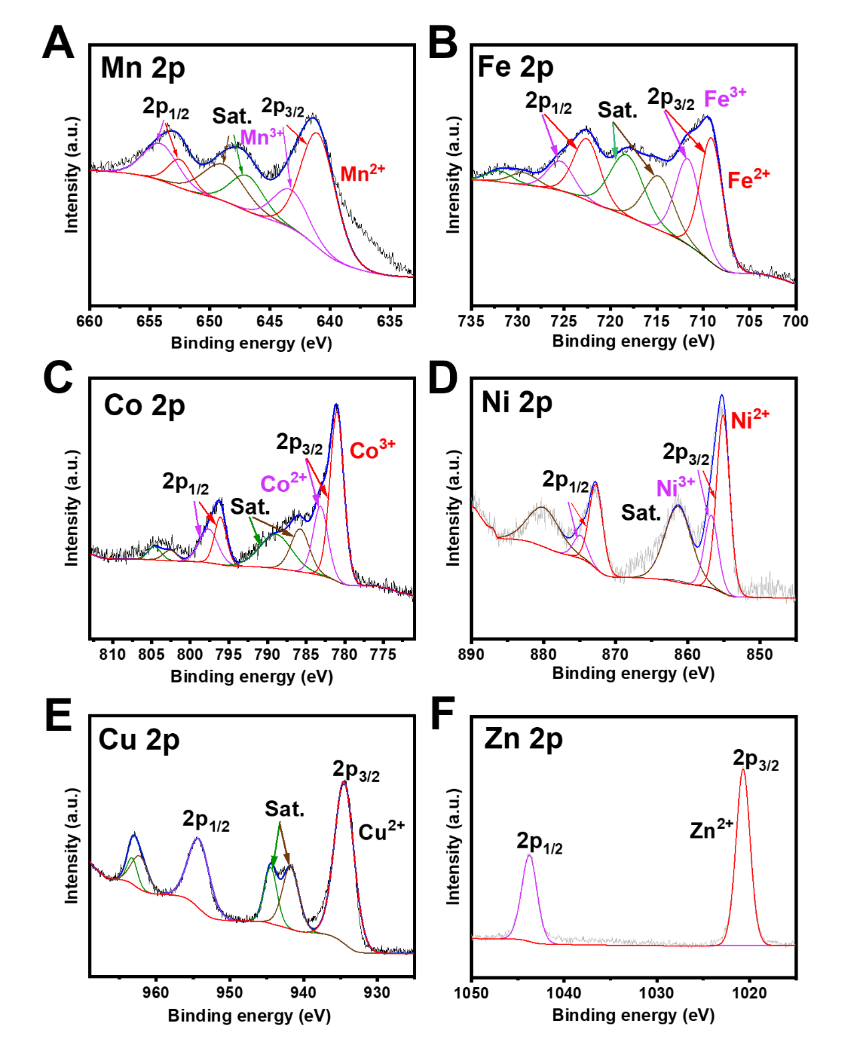


**Figure S4.** XPS spectra of A) Mn 2p, B) Fe 2p, C) Co 2p, D) Ni 2p, E) Cu 2p, and F) Zn 2p for HEO.

In the high-resolution Mn 2p spectrum, the peaks located at 654.10 and 652.50 eV were assigned to Mn^3+^ and Mn^2+^ in Mn 2p_1/2_, while the peak presented at 643.29 and 641.01 eV was on tributed to Mn^3+^ and Mn^2+^ in Mn 2p_3/2_ (Figure S4A). In the high-resolution Fe 2p spectrum, the peaks located at 725.40 and 722.54 eV were assigned to Fe^3+^ and Fe^2+^ in Fe 2p_1/2_, while the peak presented at 711.65 and 709.10 eV was contributed to Fe^3+^ and Fe^2+^in Fe 2p_3/2_ (Figure S4B). In the high-resolution Co 2p spectrum, the binding energies at 785.78 and 802.61 eV correspond to the Co 2p_3/2_ and Co 2p_1/2_ of Co^2+^, respectively, and the binding energies at 783.14 and 797.63 eV correspond to the Co 2p_3/2_ and Co 2p_1/2_ of Co^3+^, respectively (Figure S4C). In the high-resolution Ni 2p spectrum, the binding energies at 855.1 and 872.73 eV can be assigned to the Ni 2p_3/2_ and Ni 2p_1/2_ of Ni^2+^, respectively, and the binding energies at 857.75 and 874.90 eV can be assigned to the Ni 2p_3/2_ and Ni 2p_1/2_ of Ni^3+^ (Figure S4D). In the Cu 2p spectrum, the binding energies at 934.50 and 954.22 eV correspond to the Cu 2p_3/2_ and Cu 2p_1/2_ of Cu^2+^ (Figure S4E). In the Zn 2p spectrum, the binding energies at 1021.70 and 1043.75 eV correspond to the Zn 2p_3/2_ and Zn 2p_1/2_ of Zn^2+^ (Figure S4F).


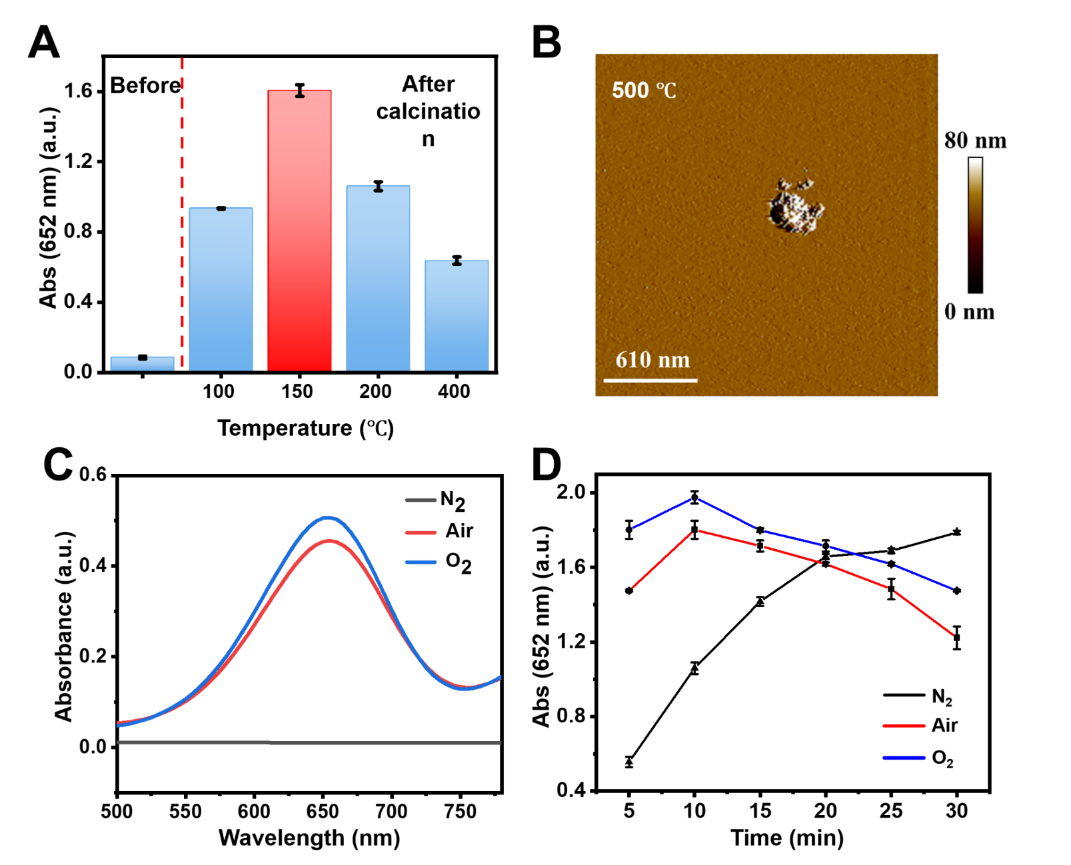


**Figure S5.** A) The catalytic activity of HEO before and after calcination at different temperatures from 100-400 ℃. B) AFM image of HEO after calcined at 500 ℃ for 1 h. Scale bar: 610 nm. C) HEO before and after calcination at Comparison of UV-Vis absorbance spectra of HEO/TMB system in NaAc-HAc buffer (pH = 4.00) for 5 min under N_2_ (black line), O_2_ (blue line), and air (red line) environment. D) The relationship between A_652_ and time for the HEO/TMB/H_2_O_2_ system under N_2_ (black line), O_2_ (blue line), and air (red line) conditions.


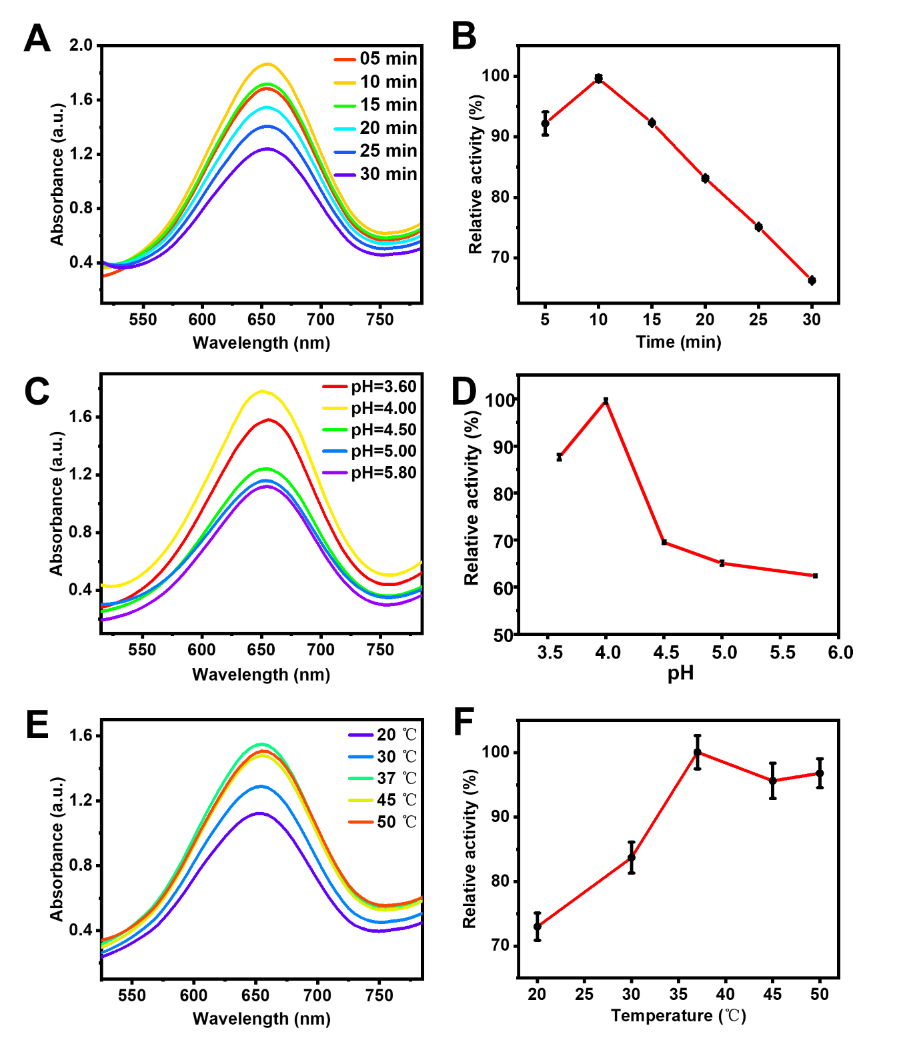


**Figure S6.** The optimization condition for HEO/TMB/H_2_O_2_ system including of A) and B) reaction time within 5-30 min, C) and D) pH between 3.60-5.50, and E) and F) temperature from 20 to 50 ℃. The absorbance spectra (left) and corresponding relationship between relative activity and reaction parameters (right). The highest point in the relative activity was defined as 100 %.


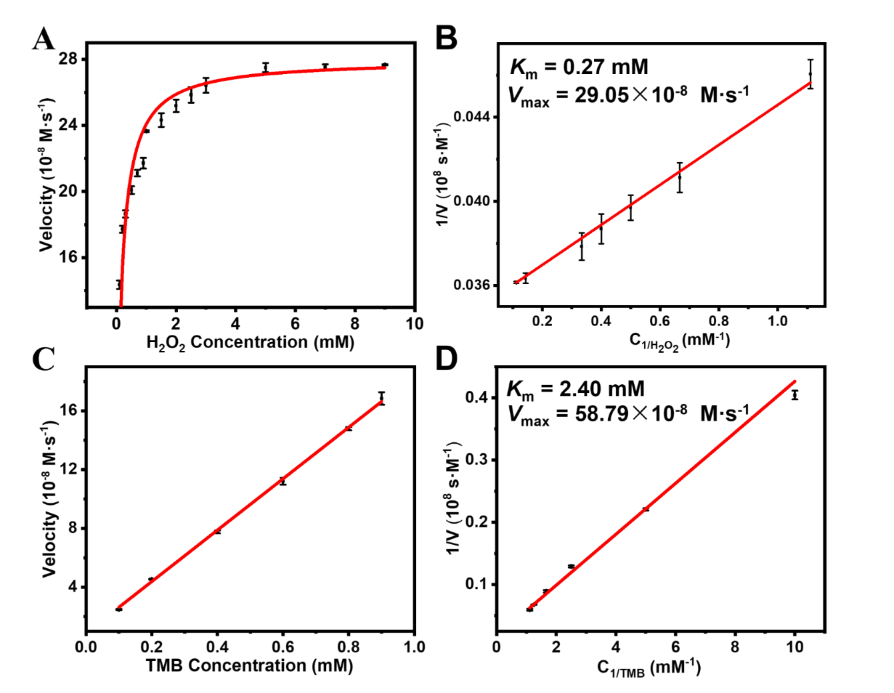


**Figure S7.** Steady-state kinetic assay of HEO towards A) and B) H_2_O_2_ from 0.90 mm to 9 mm and C) and D) TMB from 0.10 mm to 0.90 mm.


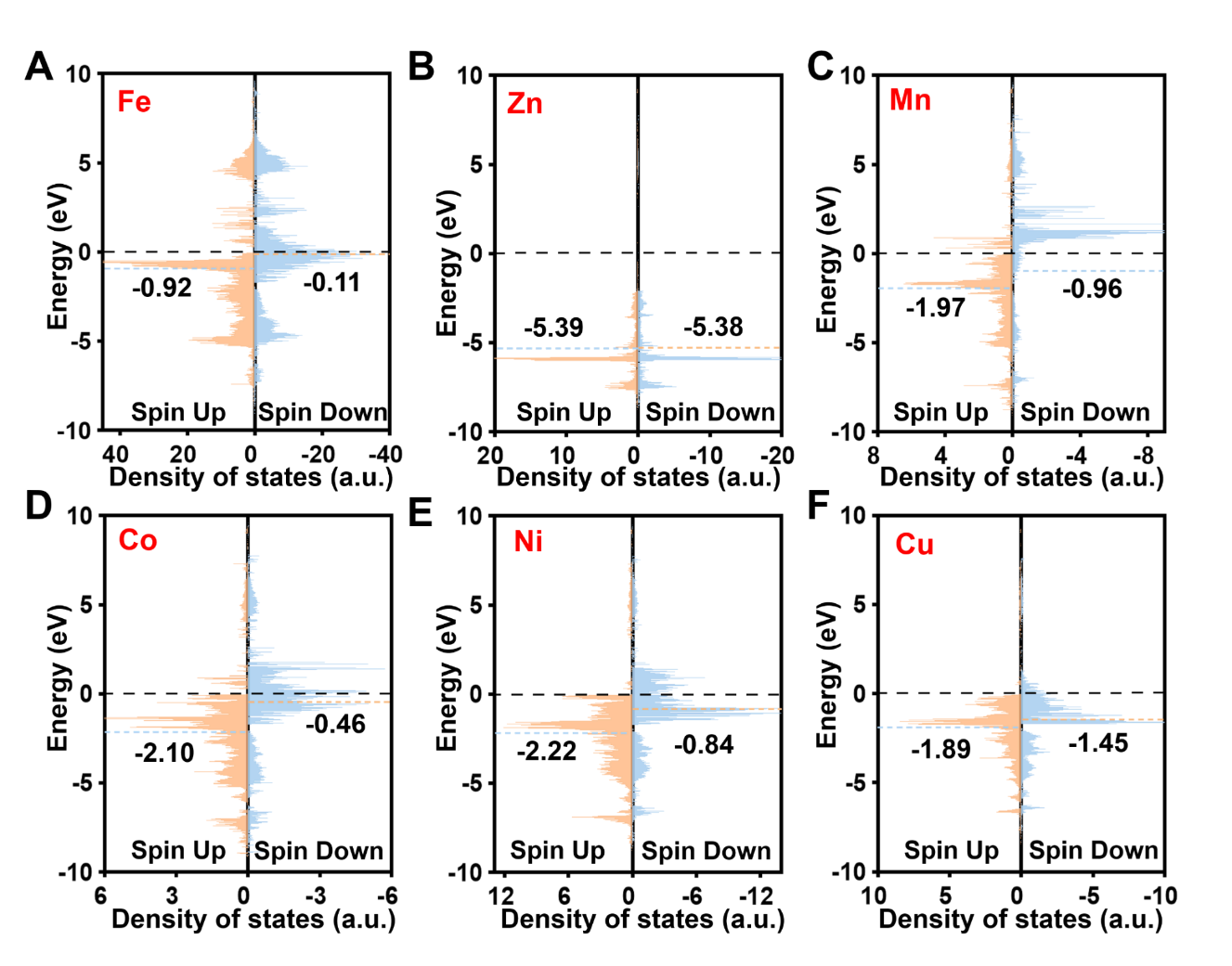


**Figure S8.** The PDOS and *d*-band centers (including spin up and spin down) for A) Fe, B) Zn, C) Mn, D) Co, E) Ni, and F) Cu in the HEO.


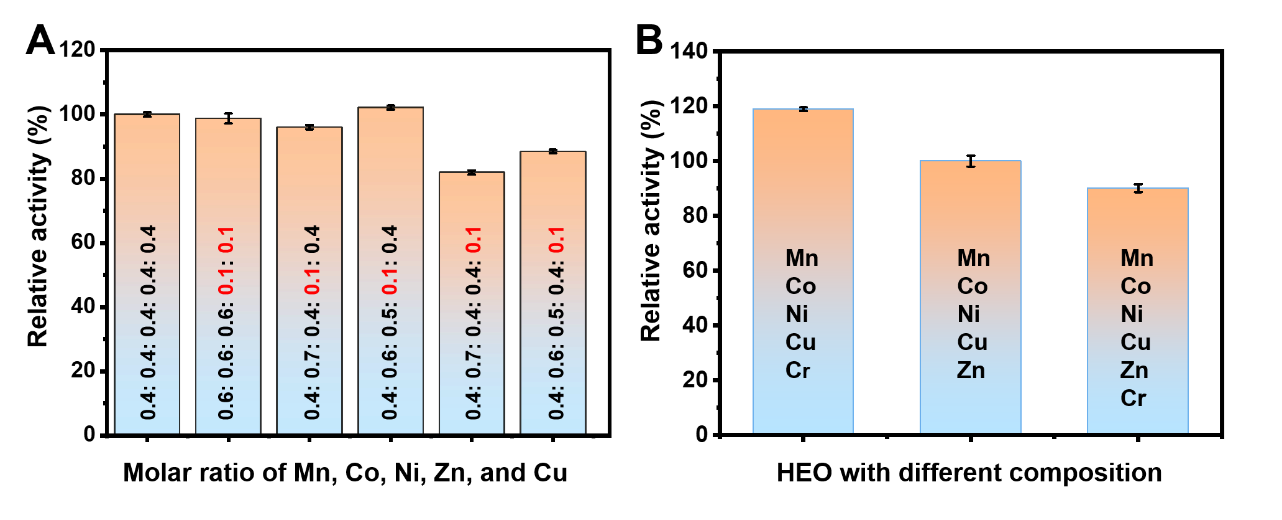


**Figure S9.** A) The catalytic activity of HEO with different molar ratios for five metal precursors (Mn:Co:Ni:Zn:Cu) while keeping the total metal concentration constant (*n* = 3). B) Comparison of catalytic activity of Cr-containing HEO such as MnCoNiCuCr-HEO and MnCoNiCuZnCr-HEO (*n* = 3).


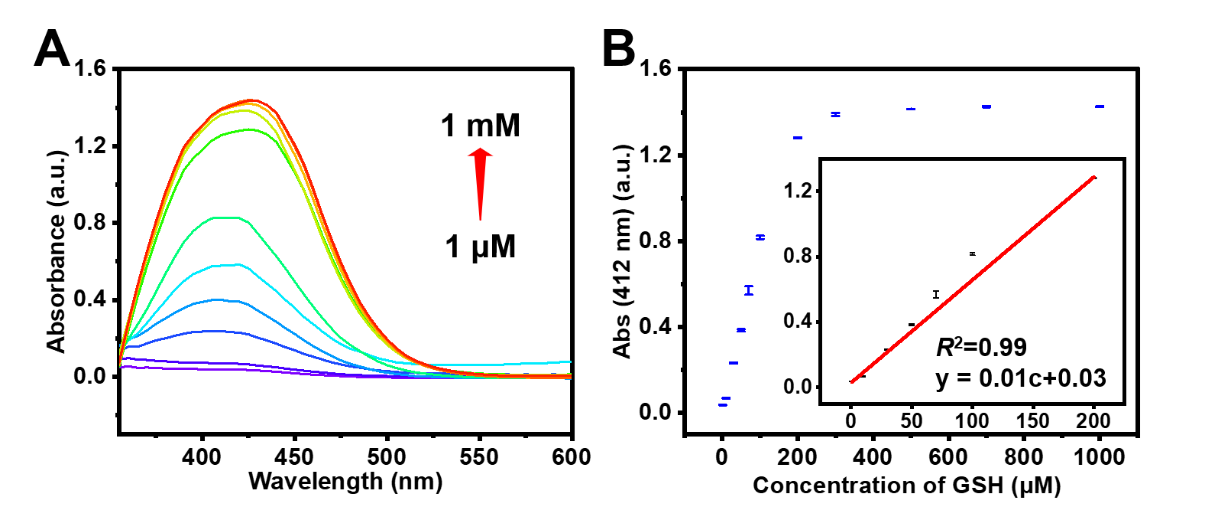


**Figure S10.** A) UV-Vis absorption spectra of the HEO/DTNB system with GSH concentrations ranging from 1 μm to 1 mm. B) The correlation between absorbance at 412 nm (A_412_, TNB) and GSH concentrations (*n* = 3). Inset is the fitted calibration curve of A_412_ versus GSH concentration.


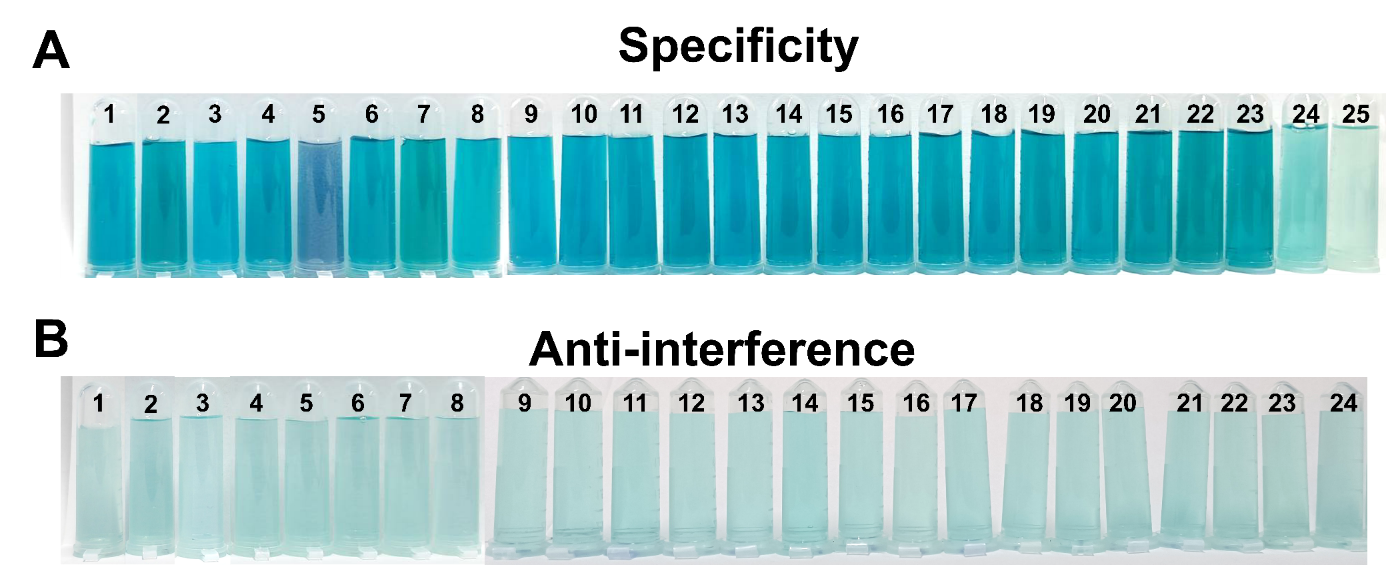
**Figure S11.** Color variation of the HEO/TMB/H_2_O_2_ based reaction systems in the absence A) and presence B) of GSH. The tubes includes blank (1), Na^+^ (2), Zn^2+^ (3), Mg^2+^ (4), Cu^2+^ (5), K^+^ (6), Ca^2+^ (7), AA (8), Arg (9), Glu (10), Asp (11), Gly (12), Val (13), Asn (14), Lys (15), His (16), Thr (17), Ser (18), BSA (19), Tyr (20), Met (21), Phe (22), uric acid (23), Cys (24), and GSH (25). *Note*: Cys, GSH, 50 μm; AA, 0.20 mm; others, 2 mm.


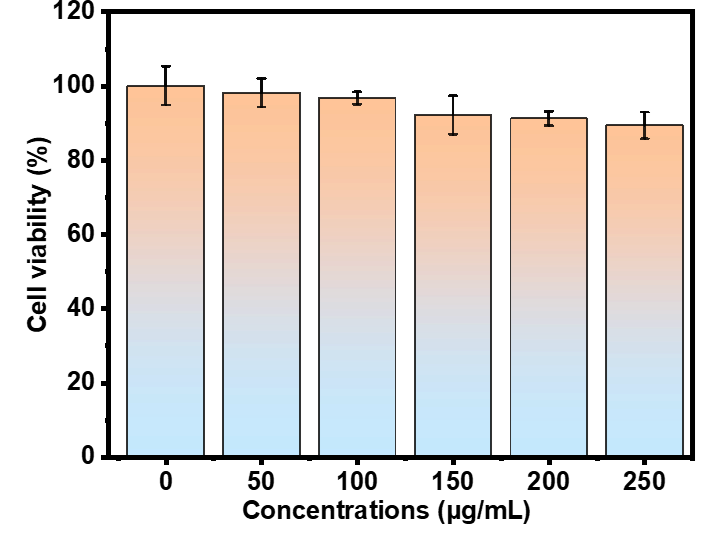


**Figure S12.** Comparison of the cell viability of HEK293 cells after incubating with different concentrations of HEO between 0-250 μg·mL^-1^ based on MTT assay (*n* = 3).


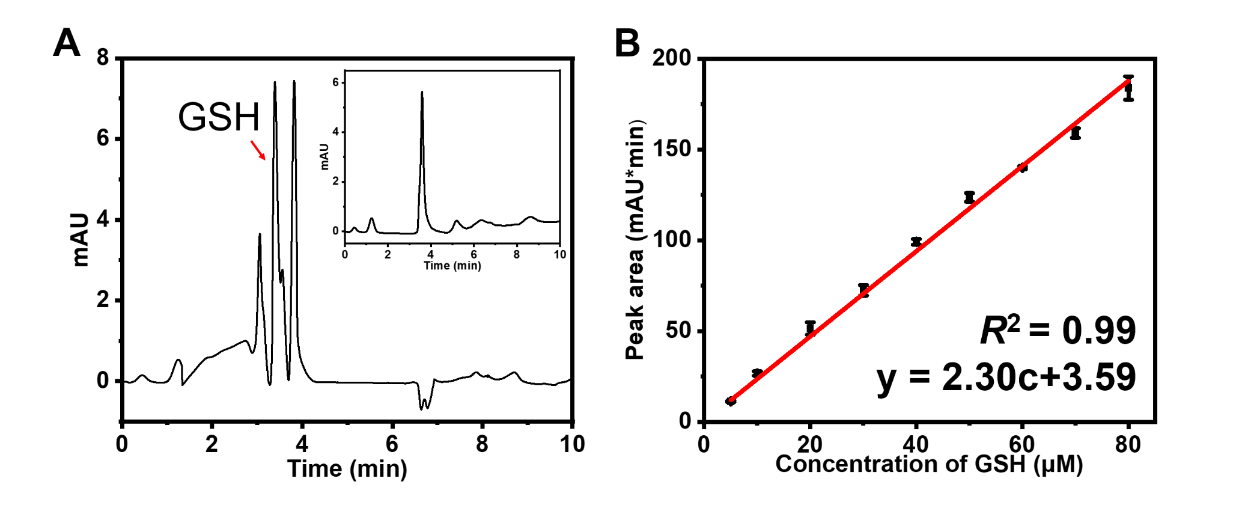


**Figure S13.** A) HPLC chromatogram of the diluted goat serum sample and the peak indicated by the red arrow corresponds to GSH with a retention time of approximately 3.40 min. The inset shows the chromatogram of 20 μm standard GSH, which exhibits a retention time of approximately 3.58 min. Chromatographic conditions: 50 mm potassium dihydrogen phosphate (pH 2.50): methanol = 90: 10, 0.6 mL/min, 30 ℃, UV 210 nm. B) The standard curve for GSH determination using HPLC method (*n* = 3).


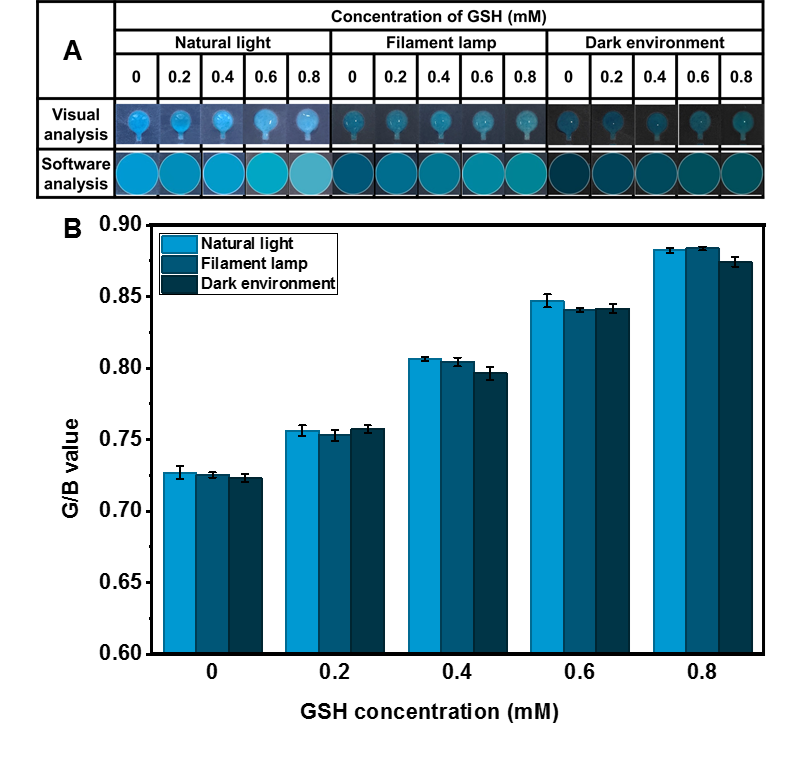


**Figure S14**. A) Color transitions and B) G/B values of the HEO/TMB/H_2_O_2_ reaction solutions with different GSH concentrations between 0.0-0.8 mm under three conditions including natural light (light blue), filament lamp (medium blue), and complete darkness (dark blue) (*n* = 3).

**Table S1.** Comparison of *K*_m_, *V*_max_, and *K*_cat_ for HEO with other reported nanozymes.

| **Nanozymes** | **H_2_O_2_** | | | **TMB** | | | **Reference** |
| --- | --- | --- | --- | --- | --- | --- | --- |
|  | ***K*_m_ (mm)** | ***V*_max_(10^−8^ M·s^−1^)** | ***K*_cat_ (s^-1^)** | ***K*_m_ (mm)** | ***V*_max_(10^−8^ M·s^−1^)** | ***K*_cat_ (s^-1^)** |  |
| MnFeCoNiCu transition metal high-entropy nanozymes (HEzymes) | 0.60 | 16.62 | 3.23×10^8^ | 0.07 | 6.26 | 1.22×10^8^ | [1] |
| Iron-doped ZIF-8 nanoparticle, loaded with hypoxia-enhanced anti-tumor drug (Fe-ZT) | 79.40 | 22.50 | --- | --- | --- | --- | [2] |
| High entropy two-dimensional layered double hydroxide (AlFeCoCuZn HE-LDH) | 18.13 | 14.73 | --- | 280.70 | 16.80 | --- | [3] |
| Mussel-inspired Fe-based Tannic acid Nanozyme (FTAN) | 3.50 | 13.11 | --- | 0.15 | 16.42 | --- | [4] |
| Selenium nanoparticles-MgFe-LDH nanosheets-bioactive glass scaffold (BGS@LDH/Se) | 0.82 | 3.67 | --- | --- | --- | --- | [5] |
| Zinc (Zn) doped Fe-MOFs (ZFMs) | 0.26 | 4.98 | --- | 1.67 | 7.75 | --- | [6] |
| Ultra-small high-entropy alloy nanoparticles (PtPdRuRhIr US-HEANPs) | 4.09 | 18.82 | --- | 0.03 | 24.57 | --- | [7] |
| CeO_2_Mn_1.08_O_x_ nanoclusters | 10.74 | --- | --- | --- | --- | --- | [8] |
| Red light carbon dots nanozyme and glucose oxidase (FG) | 8.13 | 19.13 | --- | 0.47 | 18.68 | --- | [9] |
| FeCuAgCeGd–HEAzyme | 756 | 9.65 | --- | 6.60 | 16.9 | --- | [10] |
| Horseradish peroxidase (HRP) | 3.70 | 8.71 | 3.48×10^3^ | 0.43 | 10 | 4×10^3^ | [1] |
| Palladium (Pd) seeds | 70.30 | 14 | 1.30×10^3^ | 0.05 | 38 | 3.60×10^3^ | [11] |
| Ru frames | 31.80 | 7.41 | 6.98×10^3^ | 0.06 | 13.40 | 1.26×10^4^ | [12] |
| HEO (MnCoNiCuZnFe) | 0.27 | 29.05 | 1.76×10^4^ | 2.40 | 58.79 | 3.56×10^4^ | This work |

**Table S2.** Comparison of response performance of reported nanomaterials for the colorimetric detection of GSH.

| Nanomaterials | LOD  (μm) | Linear range (μm) | Reference |
| --- | --- | --- | --- |
| Hemin-functionalized peptide nanotubes (hemin-PNTs) | 0.51 | 1.00-30 | [13] |
| Ferroelectric BaTiO_3_ nanoparticles (BTO NPs) | 0.20 | 0.50-20 | [14] |
| Ultrathin FeS nanosheets (NSs) | 0.14 | 2-50 | [15] |
| Pd nanoparticles-decorated Zn/Co zeolitic imidazole framework nano composite (Zn/Co-ZIF@PdNPs) | 0.12 | 0.30-12 | [16] |
| Nitrogen-doped carbon nanofiber (α-Co@NCNF) nanozyme | 0.03 | 0.10-20 | [17] |
| Novel ultrathin two-dimensional (2D) COF (termed as TTPA-COF) nanosheets | 0.50 | 0.50-40 | [18] |
| Nitrogendoped reduced graphene oxide (MoS_2_/N-rGO) | 0.12 | 2-15 | [19] |
| Manganese phthalocyanine @ graphene nanoplatelets (MnPc@GNP). | 2.42 | 1-30 | [20] |
| HEO (MnCoNiCuZnFe) | 0.07 | 0.10-70 | This work |

**Table S3.** Recovery test for GSH in diluted goat serum based on the proposed HEO system in comparison with HPLC.

| Sample | GSH spiked (μm) | HPLC | | | HEO | | |
| --- | --- | --- | --- | --- | --- | --- | --- |
|  |  | **Found (μm)** | **Recovery (%)** | **RSD (%)** | **Found (μm)** | **Recovery (%)** | **RSD (%)** |
| 1 | 5.00 | 5.05 | 101.05 | 0.94 | 4.97 | 100.80 | 2.51 |
|  | 10.00 | 9.97 | 99.66 | 1.25 | 9.69 | 98.30 | 2.47 |
|  | 20.00 | 19.89 | 99.40 | 1.12 | 19.99 | 100.65 | 1.25 |
|  | 30.00 | 29.74 | 99.11 | 1.16 | 31.04 | 102.57 | 0.38 |
| 2 | 5.00 | 5.11 | 102.27 | 1.07 | 5.06 | 101.13 | 1.69 |
|  | 10.00 | 10.04 | 100.36 | 1.26 | 10.24 | 102.37 | 1.40 |
|  | 20.00 | 19.96 | 99.81 | 0.08 | 20.03 | 100.13 | 1.00 |
|  | 30.00 | 29.89 | 99.62 | 0.48 | 29.66 | 98.88 | 0.97 |
| 3 | 5.00 | 4.95 | 99.05 | 1.12 | 4.99 | 99.77 | 2.06 |
|  | 10.00 | 10.23 | 102.27 | 1.44 | 9.98 | 99.78 | 0.95 |
|  | 20.00 | 20.24 | 101.18 | 1.07 | 19.74 | 98.70 | 0.73 |
|  | 30.00 | 30.38 | 101.27 | 0.26 | 30.35 | 101.15 | 0.47 |

**Reference**

[1] J. Feng, X. Yang, T. Du, L. Zhang, P. Zhang, J. Zhuo, L. Luo, H. Sun, Y. Han, L. Liu, Y. Shen, J. Wang, W. Zhang, Transition metal high‐entropy nanozyme: multi‐site orbital coupling modulated high‐efficiency peroxidase mimics, *Adv. Sci.* **2023**, *10* (33), e2303078.

[2] F. Li, P. Zhu, B. Zheng, Z. Lu, C. Fang, Y. Fu, X. Li, A customized biohybrid presenting cascade responses to tumor microenvironment, *Adv. Mater.* **2024**, *36* (30), e2404901.

[3] C. Wang, F. Yuan, Z. Yan, T. Zhang, C. Fu, Y. Li, G. Dai, H. Kim, S. Xia, L. Yu, S. Debnath, W. Ren, J. Shu, M. Qiu, J.S. Kim, High entropy 2D layered double hydroxide nanosheet toward cascaded nanozyme-initiated chemodynamic and immune synergistic therapy, *J. Am. Chem. Soc.* **2024**,*147* (1), 136.

[4] S. Liu, R. Shu, J. Ma, L. Dou, W. Zhang, S. Wang, Y. Ji, Y. Li, J. Xu, D. Zhang, M. Zhu, Y. Song, J. Wang, Mussel-inspired Fe-based tannic acid nanozyme: a renewable bioresource-derived high-affinity signal tag for dual-readout multiplex lateral flow immunoassay, *Chem. Eng. J.* **2022**, *446*(4), 137382.

[5] Y. Bian, K. Zhao, T. Hu, C. Tan, R. Liang, X. Weng, A Se nanoparticle/MgFe-LDH composite nanosheet as a multifunctional platform for osteosarcoma eradication, antibacterial and bone reconstruction, *Adv. Sci.* **2024**, *11*(33), 2403791.

[6] D. Zhong, Y. Zuo, Y. Shi, P. Zhang, Y. Xu, B. Li, Right once for all: Zinc-modulated highly stable iron-based ROS generator under physiological conditions for promoting bacteria-infected wound healing, *C**hem. Eng. J.* **2023**, *460*, 141837.

[7] Y. Ai, M. He, H. Sun, X. Jia, L. Wu, X. Zhang, H. Sun, Q. Liang, Ultra-small high-entropy alloy nanoparticles: efficient nanozyme for enhancing tumor photothermal therapy, *Adv. Mater.* **2023**, *35* (23), e2302335.

[8] Q. Qiao, Z. Liu, F. Hu, Z. Xu, Y. Kuang, C. Li, A novel Ce-Mn heterojunction-based multi-enzymatic nanozyme with cancer-specific enzymatic activity and photothermal capacity for efficient tumor combination therapy, *Adv. Funct. Mater.* **2024**, *35* (6), 2414837.

[9] L. Song, Q. Zhao, S. Feng, Y. He, Y. Li, S. Wang, J. Zhang, Depolymerizable enzymatic cascade nanoreactor for self-enhancing targeting synergistic tumor therapy, *Adv. Funct. Mater.* **2024**, *35* (4), 2414121.

[10] R. Sheng, Y. Liu, T. Cai, R. Wang, G. Yang, T. Wen, F. Ning, H. Peng, Ultrafine FeCuAgCeGd-based high-entropy nanozyme: Preparation, catalytic mechanism, and point–of–care detection of dopamine in human serum, *Chem. Eng. J.* **2024**, 485, 149913.

[11] H. Ye, J. Mohar, Q. Wang, M. Catalano, M. Kim, X. Xia, Peroxidase-like properties of Ruthenium nanoframes, *Sci. Bull.* **2016**, *61* (22) 1739.

[12] C. Verma, P. Singh, R. Ojha, R. Prakash, Hierarchically porous 2D carbon from bio-waste: a sustainable, rapid, and efficient oxidase mimic for the colorimetric detection of ascorbic acid, *Mater. Adv.* **2022**, *3* (6), 2749.

[13] S. Xiang, X. Long, Q. Tu, J. Feng, X. Zhang, G. Feng, L. Lei, Self-assembled, hemin-functionalized peptide nanotubes: an innovative strategy for detecting glutathione and glucose molecules with peroxidase-like activity, *Nano Converg.* **2023**, *10* (1), 7.

[14] D. Yang, J. Liu, W. Hu, Y. Xiao, H. Chen, Y. Long, H. Zheng, Nano-ferroelectric oxidase mimics for colorimetric detection of glutathione, *Sensor. Actuat. B-Chem.* **2023**, *393*.

[15] Y. Duan, Q. Li, P. He, Y. Li, J. Song, J. Wang, J. Liu, J. Zhou, F. Chen, Z. Huang, J. Sun, Y. Zhang, Z. Luo, Ultrathin FeS nanosheets with high chemodynamic activity for sensitive colorimetric detection of H_2_O_2_ and glutathione, *Chinese Chem. Lett.* **2022**, *33* (6), 3217.

[16] F. Li, Y. Liu, W. Jia, R. Ma, W. Zhang, L. Shang, X. Li, H. Wang, L. Jia, Pd nanoparticles on Zn/Co zeolitic imidazolate frameworks: a H_2_O_2_-free oxidase mimic for dual colorimetric and fluorescent detection of glutathione, *Sensor. Actuat. B-Chem.* **2025**, *429*, 137294.

[17] Y. Xia, F. Shi, R. Liu, H. Zhu, K. Liu, C. Ren, J. Li, Z. Yang, In situ electrospinning MOF-derived highly dispersed alpha-cobalt confined in nitrogen-doped carbon nanofibers nanozyme for biomolecule monitoring, *Anal. Chem.* **2024**, *96* (3), 1345.

[18] Y. Peng, M. Huang, L. Chen, C. Gong, N. Li, Y. Huang, C. Cheng, Ultrathin covalent organic framework nanosheet-based photoregulated metal-free oxidase-like nanozyme, *Nano Research.* **2022**, *15* (10), 8783.

[19] L. Wang, B. Li, Z. You, A. Wang, X. Chen, G. Song, L. Yang, D. Chen, X. Yu, J. Liu, C. Chen, Heterojunction of vertically arrayed MoS_2_ nanosheet/N-doped reduced graphene oxide enabling a nanozyme for sensitive biomolecule monitoring, *Anal. Chem.* **2021**, *93* (32), 11123.

[20] D. Baruah, A. Thakur, E. Roy, K. Roy, S. Basak, D. Neog, H. Bora, R. Konwar, V. Chaturvedi, M. Shelke, M. Das, Atomically dispersed manganese on graphene nanosheets as biocompatible nanozyme for glutathione detection in liver tissue lysate using microfluidic paper-based analytical devices, *ACS Appl. Mater. Interfaces.* **2023**, *15* (41), 47902.
